# Proton extrusion during oxidative burst in microglia exacerbates pathological acidosis following traumatic brain injury

**DOI:** 10.1002/glia.23926

**Published:** 2020-10-22

**Authors:** Rodney M. Ritzel, Junyun He, Yun Li, Tuoxin Cao, Niaz Khan, Bosung Shim, Boris Sabirzhanov, Taryn Aubrecht, Bogdan A. Stoica, Alan I. Faden, Long‐Jun Wu, Junfang Wu

**Affiliations:** ^1^ Department of Anesthesiology and Center for Shock Trauma and Anesthesiology Research (STAR), University of Maryland School of Medicine Baltimore Maryland USA; ^2^ University of Maryland Center to Advance Chronic Pain Research, University of Maryland Baltimore Maryland USA; ^3^ Department of Neurology Mayo Clinic Rochester Minnesota USA

**Keywords:** acidosis, chronic neurodegeneration, microglia, neuroinflammation, traumatic brain injury

## Abstract

Acidosis is among the least studied secondary injury mechanisms associated with neurotrauma. Acute decreases in brain pH correlate with poor long‐term outcome in patients with traumatic brain injury (TBI), however, the temporal dynamics and underlying mechanisms are unclear. As key drivers of neuroinflammation, we hypothesized that microglia directly regulate acidosis after TBI, and thereby, worsen neurological outcomes. Using a controlled cortical impact model in adult male mice we demonstrate that intracellular pH in microglia and extracellular pH surrounding the lesion site are significantly reduced for weeks after injury. Microglia proliferation and production of reactive oxygen species (ROS) were also increased during the first week, mirroring the increase in extracellular ROS levels seen around the lesion site. Microglia depletion by a colony stimulating factor 1 receptor (CSF1R) inhibitor, PLX5622, markedly decreased extracellular acidosis, ROS production, and inflammation in the brain after injury. Mechanistically, we identified that the voltage‐gated proton channel Hv1 promotes oxidative burst activity and acid extrusion in microglia. Compared to wildtype controls, microglia lacking Hv1 showed reduced ability to generate ROS and extrude protons. Importantly, Hv1‐deficient mice exhibited reduced pathological acidosis and inflammation after TBI, leading to long‐term neuroprotection and functional recovery. Our data therefore establish the microglial Hv1 proton channel as an important link that integrates inflammation and acidosis within the injury microenvironment during head injury.

## Main Points

- Head injury causes chronic acidosis in microglia and the surrounding lesion site
- Microglia depletion attenuated brain acidosis and ROS levels
- The proton channel Hv1 promoted extracellular release of H+ and ROS from microglia and worsened outcome

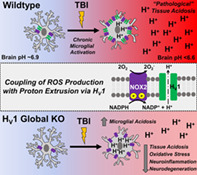

## 1 INTRODUCTION

After ischemia or moderate/severe brain trauma, tissue acidosis is a common characteristic of brain damage, contributing to the prolongation of coma and long‐term neurologic deficit (Clausen et al., 2005; Marmarou, 1992; Marmarou et al., 1993). The lack of blood supply to affected regions causes a shortage of oxygen that results in increased anaerobic glycolysis. This metabolic crisis occurs frequently after traumatic brain injury (TBI; Brooks & Martin, 2014; Carre et al., 2013; Timofeev, Nortje, Al‐Rawi, Hutchinson, & Gupta, 2013), and causes the accumulation of lactic acid, protons (i.e., H^+^), and carbonic acid, decreasing extracellular pH. Although physiological brain pH is maintained at ~7.0–7.4, below this buffering threshold, acidosis can become pathological by affecting ion channel and receptor activity, glutamate reuptake, endoplasmic reticulum stress, mitochondrial dysfunction, glial cell activation, and neuronal apoptosis (de Ceglia et al., 2015; Dodge et al., 2013).

Although brain tissue acidosis has long been recognized as an important pathological feature of TBI, little is known regarding the cellular and molecular mechanisms involved. Based upon correlational clinical studies, lactic acidosis was implicated as a key mechanism, as brain lactate concentrations are inversely correlated with brain pH after head injury and appear to reliably predict both short‐term and long‐term clinical outcomes (Clausen et al., 2005; Ellingson et al., 2019; Gupta et al., 2004; Rehncrona, 1985). But the current understanding of TBI‐induced acidosis and its underlying mechanisms is limited. More specifically, the potential role of non‐neuronal cells and lactate‐independent mechanisms in posttraumatic acidosis after injury have been little examined.

Brain acidosis is associated with neuroinflammation after TBI. However, whether CNS‐resident microglia regulate the pH of their environment is unclear (Tyrtyshnaia et al., 2016). Low pH induces pro‐inflammatory responses in CNS‐resident microglia (Erra Diaz, Dantas, & Geffner, 2018). In the activated state, phagocytes such as microglia increase respiratory burst activity which generates reactive oxygen species (ROS), causing oxidative stress. During this process, the phagocyte nicotinamide adenine dinucleotide phosphate (NADPH) oxidase (NOX2) induces superoxide anion at the expense of cytosolic NADPH, generating free protons (H^+^) which leads to intracellular acidification (Clark, 2016; El Chemaly, Nunes, Jimaja, Castelbou, & Demaurex, 2014). Yet the extent to which microglia play a role in brain acidosis is unknown and the functional importance of proton transport regulation in tissue acidosis has also yet to be explored. Moreover, the relationship between NOX, ROS, and pH led us to surmise that the voltage‐gated proton channel Hv1 might be involved in brain acidosis. Hv1 supports NOX activity by exporting excess protons generated during ROS production (PMID: 28961043). An important acid extruding ion channel (Musset et al., 2008; Seredenina, Demaurex, & Krause, 2015), Hv1 is predominantly expressed by microglia within the CNS (Ramsey, Ruchti, Kaczmarek, & Clapham, 2009; L. J. Wu, 2014; L. J. Wu et al., 2012). Prior work provides a rationale for Hv1 as a potential therapeutic target for the treatment of ischemic stroke (Tian et al., 2016; L. J. Wu et al., 2012). However, neither the precise cellular mechanisms underlying its actions nor its potential role in the pathophysiology of TBI have been addressed. We demonstrate that activated microglia increase the extrusion of protons and ROS across the plasma membrane into the extracellular space after experimental TBI, contributing to pathological brain acidosis that leads to chronic neurodegeneration and related functional deficits. Moreover, we show that microglial voltage‐gated proton channel Hv1 is a key mechanism that can be targeted to attenuate acidosis, oxidative stress, and neuroinflammation.

## 2 MATERIALS AND METHODS

### 2.1 Animals and controlled cortical impact injury

All surgical procedures and animal experiments were performed according to protocols approved by the University of Maryland School of Medicine Institutional Animal Care and Use Committee (IACUC). Adult male C57BL/6 mice at 10–12 weeks old (22–25 g) were purchased from Jackson Laboratories. Heterozygous Hv1 mice breeders were obtained from Dr. Long‐Jun Wu's laboratory at Mayo Clinic, Rochester, MN and maintained in the UMB animal facility. Controlled cortical impact (CCI) was performed with a TBI‐0310 impactor (Precision Systems & Instrumentation, VA). Briefly, under isoflurane anesthetization, a 10‐mm midline incision was made over the skull, the skin and fascia were retracted, and a 4‐mm craniotomy was made on the central aspect of the left parietal bone of mice under surgical anesthesia (Ritzel et al., 2020). A moderate injury was induced by a 3.5‐mm diameter tip with impact velocity of 4 m/s and a deformation depth of 1.2 mm. Sham mice went through the same procedures without craniotomy and impaction. After CCI, mice were assigned to a group according to a randomized block experimental design. The number of mice in each study was indicated in the figure legends. The surgical procedures were performed by the same investigators blinded with mice genotypes or drug treatments.

### 2.2 | Extracellular pH recordings

**FIGURE 1:**
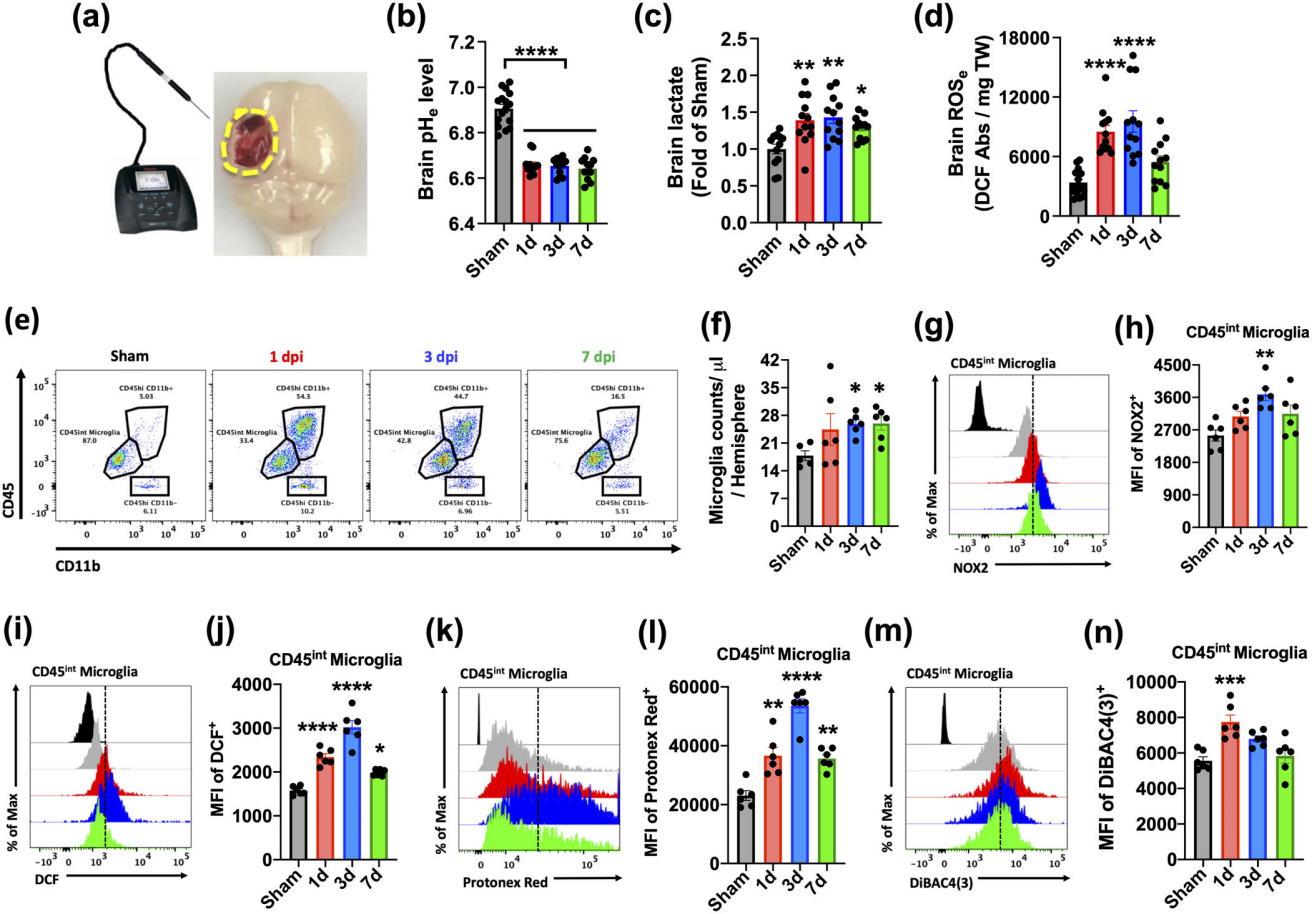
Acute TBI induced intracellular microglial acidosis and extracellular brain acidosis. (a) Diagram of experimental procedure for brain pH recordings. A pH meter micro‐electrode probe was inserted at several points around the ipsilateral sham and perilesional cortex and the median pH value of 10 recordings was taken. (b) Extracellular brain pH (pH_e_) was measured in sham mice and Day 1, 3, and 7 after TBI (*n* = 16, 12, 12, and 12/group). (c) Extracellular brain lactate concentrations are shown as a relative fold change to sham control during the first week of injury (*n* = 12, 12, 12, and 12/group). (d) Extracellular reactive oxygen species (ROS_e_) levels were increased at Day 1 and 3 relative to sham control (*n* = 12, 12, 12, and 12/group). (e) A representative dot plot of immune cells in the brain of sham and injured mice at Day 1, 3, and 7 after TBI is shown. (f) Quantification of CD45^int^CD11b^+^Ly6C^−^ microglia counts in the ipsilateral hemisphere during the first week of injury. (g) A representative histogram shows the relative mean fluorescence intensity of NOX2^+^ microglia. (h) Mean fluorescence intensity of NOX2 expression in microglia was quantified. (i) A representative histogram shows the relative mean fluorescence intensity of DCF^+^ microglia. (j) Increased microglial ROS production was found at Day 1, 3, and 7 after TBI compared to sham. (k) A representative histogram shows the relative mean fluorescence intensity of Protonex Red^+^ microglia. (l) Decreased cytosolic pH in microglia was evident at Day 1, 3, and 7 after TBI compared to sham. (m) A representative histogram shows the relative mean fluorescence intensity of DiBAC4(3)^+^ microglia. (n) Increased plasma membrane depolarization in microglia was found at day 1 and 3 after TBI compared to sham. Data for (b–d) are representative of two independent experimental cohorts. For all flow cytometry experiments, *n* = 6/group. For all flow cytometry histograms, fluorescence minus one (FMO) controls are shown in black, time points are color coded according to bar graph, and a vertical fiducial line is included for reference. Abbreviations: Abs absorbance, Max Maximum, MFI mean fluorescence intensity, mg milligram, TW tissue weight. All data were analyzed by one‐way ANOVA using Dunnett's multiple comparison test to determine differences between sham and each time point postinjury (*p < .05, **p < .01, ***p < .001, and ****p < .0001) [Color figure can be viewed at wileyonlinelibrary.com]

On various time‐points (1, 3, 7, 14, and 28 days) after CCI, anesthetized mice using Euthasol were intracardially perfused with 40 ml of cold normal saline. After removal of the brain on ice, extracellular pH of the cerebral cortex was obtained with the Orion Star A211 benchtop pH meter (Cat# STARA2110, Thermo fisher Scientific) using a micro pH electrode with glass body and needle tip (Cat# 9863BN, Thermo fisher Scientific) that has a measurement accuracy up to 0.02 connected to the pH meter. Before each experiment, a three‐point calibration (pH 4.0, pH 7.0, and pH 10.0) was performed on the pH meter to ensure accuracy of the readings. In order to avoid the confounding factor of blood within the lesion center and increase accuracy, we took readings from 10 different points surrounding the perilesion site of the ipsilateral cortex (Figure 1a) and the average value was taken as the extracellular pH for each animal. For sham animals, readings were taken from similar areas of the left cerebral cortex. The micro electrode was inserted no deeper than 1–1.5 mm into the cortex tissue and remained in position until the value was stable. The micro pH electrode was cleaned with double distilled water between each mouse and cleaned thoroughly after each experiment with the pH electrode cleaning solution kit (Cat# 900020, Thermo fisher Scientific) to remove any remaining protein and lipid deposits on the micro electrode.

For recordings of blood pH, approximately 200 μl were taken from each animal and stored in an Eppendorf tube, after which the micro electrode was inserted into the liquid and suspended via its pH meter rack in the middle without touching the tube bottom or side surfaces. The average of three recordings was taken for each animal.

### 2.3 Lactate assay

For assessment of lactate acidosis within the postinjury brain, a tissue punch was taken from the ipsilateral cerebral cortex and homogenized in 600 μl of ice‐cold molecular grade water (Cat# 351‐029‐101, Quality Biological) and centrifuged at 1,500*g* for 10 min to harvest extracellular lactate samples. The L‐Lactate Assay Kit (Cat# 700510, Cayman Chemical) was used according to manufacturers' instructions and the final results were measured with a fluorescence plate reader (Synergy Hybrid, Biotek) at Ex/Em = 535/590 and presented as fold change to sham.

### 2.4 Measurement of reactive oxygen species in brain tissue

A cortical tissue punch was taken from the ipsilateral hemisphere. The samples were weighed by electronic scale, homogenized in 600 μl of ice‐cold molecular grade water, and centrifuged at 1,500*g* for 10 min as above. The supernatant was collected and temporarily kept on ice. In a 96‐well plate, 50 μl of each sample was plated in duplicate, and 150 μl of oxidative stress indicator solution (diluted in molecular grade water) was added to a final volume of 200 μl and mixed by pipet. The fluorogenic dyes, dihydrorhodamine 123 (DHR123, 1:400; Cat# D23806, Invitrogen) and H_2_DCFDA (DCF, 20.5 μM; Cat# D399, ThermoFisher Scientific) were used to measure hydroxyl, peroxyl and other ROS activity in the sample. The plate was then covered, wrapped in aluminum foil, and incubated for 30 min at 37C. Extracellular ROS were measured using a fluorescence plate reader (Synergy Hybrid, Biotek) at Ex/Em = 490/520 and presented as absorbance units normalized to either tissue weight or protein concentration (i.e., BCA assay).

### 2.5 | flow cytometry and ex vivo functional assays

Mice were perfused with 40 ml of cold PBS and the ipsilateral (i.e., craniotomy‐side) hemisphere was isolated. The olfactory bulb and cerebellum were removed, brains were halved along the interhemispheric fissure, and the ipsilateral hemisphere was placed separately in complete Roswell Park Memorial Institute (RPMI) 1,640 (Cat# 22400105, Invitrogen) medium and mechanically and enzymatically digested in collagenase/dispase (Cat# 10269638001, 1 mg/ml; Roche Diagnostics), papain (Cat# LS003119, 5 U/ml; Worthington Biochemical), 0.5 M EDTA (Cat# 15575020, 1:1000; Invitrogen), and DNAse I (Cat# 10104159001, 10 mg/ml; Roche Diagnostics) for 1 hr at 37°C on a shaking incubator (200 rpm). The cell suspension was washed twice with RPMI, filtered through a 70‐μm filter, and RPMI was added to a final volume of 5 ml/hemisphere and kept on ice. Cells were then transferred into FACS tubes and washed with FACS buffer. Cells were then incubated with Fc Block (Cat# 101320, Clone: 93; Biolegend) for 10 min on ice, and stained for the following surface antigens: CD45‐eF450 (Cat# 48–0451‐82, Clone: 30‐F11; eBioscience), CD11b‐APC/Fire™750 (Cat# 101262, Clone: M1/70; Biolegend), Ly6C‐APC (Cat# 128016, Clone: HK1.4; Biolegend), Ly6G‐AF700 (Cat# 128024, Clone: 1A8; Biolegend), and Zombie Aqua fixable viability dye (Cat# 423102, Biolegend). Cells were then washed in FACS buffer, fixed in 2% paraformaldehyde for 10 min, and washed once more prior to adding 500 μl FACS buffer. Intracellular staining for NOX2/gp91phox‐AF647 (Cat# 3889R, 1:500; Bioss Antibodies) was performed as described previously(Doran et al., 2019).

Relative changes in membrane potential, intracellular pH level, and ROS production were measured using cell‐permeant fluorescent dye probes. The membrane potential‐sensitive probe DiBAC_4_(3) can enter depolarized cells where it binds to intracellular proteins or membrane and exhibits enhanced fluorescence (10 μM; ThermoFisher Scientific). pH was measured using Protonex™ Red 600 (Cat# 21207, 5 μM; AAT Bioquest) and pHrodo™ Red AM (Cat# P35372, Invitrogen) following manufacturer's instruction. Cellular ROS were measured using dihydrorhodamine 123 (DHR123, 1:500; Invitrogen) and H_2_DCFDA (DCF, 5 μM; ThermoFisher Scientific). In brief, each dye indicator was added to RPMI media at the specified concentration and 500 μl was aliquoted into each sample, vortexed, and incubated for 30 min in a 37C water bath. Cells were then washed twice in FACS buffer, incubated in Fc Block, and stained as above.

Data were acquired on a BD LSRFortessa cytometer using FACSDiva 6.0 (BD Biosciences) and analyzed using FlowJo (Treestar Inc.). At least 5–10 million events were collected for each sample. Countbright™ Absolute Counting Beads (Invitrogen) were used to estimate cell counts per the manufacturer's instructions. Data were expressed as either cells/μl or total counts/hemisphere. Leukocytes were first gated using a splenocyte reference (SSC‐A vs. FSC‐A). Singlets were gated (FSC‐H vs. FSC‐W), and live cells were gated based on Zombie Aqua exclusion (SSC‐A vs. Zombie Aqua‐Bv510). Resident microglia were identified as the CD45^int^ CD11b^+^Ly6C^−^ population, whereas peripheral leukocytes were identified as CD45^hi^CD11b^+^ myeloid cells or CD45^hi^CD11b^−^ lymphocytes (see Figure S1 for gating strategy). Within the myeloid fraction, monocytes were identified as Ly6C^hi^Ly6G^−^ and neutrophils, Ly6C^+^Ly6G^+^. Cell type‐matched fluorescence minus one (FMO) controls were used to determine the positivity of each antibody and indicator dye (Ritzel et al., 2019).

### 2.6 The colony stimulating factor 1 receptor (CSF1R) antagonist, PLX5622 administration

PLX5622 was provided by Plexxikon Inc. (Berkley, CA) and formulated in AIN‐76A rodent chow by Research Diets Inc. (New Brunswick, NJ) at a concentration of 1,200 ppm (Spangenberg et al., 2016). According to the provider, the specialized diet was stored in a 4°C refrigerator. Mice were provided ad libitum access to PLX5622 diet or AIN‐76A chow as vehicle control. After 2 weeks on PLX5622 or vehicle chow, mice were subjected to CCI injury and examined for acidosis and inflammation by flow cytometry and qRT‐PCR.

### 2.7 Quantitative PCR

Total RNA was extracted from the perilesional ipsilateral cortex and hippocampus of sham and CCI mice with a miRNeasy isolation kit (Cat# 74104, Qiagen, Valencia, CA). Complementary DNA (cDNA) was synthesized by a Verso cDNA RT kit (Cat# AB1453B, Thermo Scientific, Pittsburg, PA) per the manufacturer's protocol. Real‐time PCR for target mRNAs was performed using TaqMan gene expression assays for cybb (NOX2), Mm01287743_m1; Cyba (p22phox) Mm00514478_m1; ITGAM (CD11b), Mm00434455_m1; TNFα, Mm00443258_m1; Il1b (IL‐1β), Mm00434228_m1; IL‐6, Mm00446190_m1; chil3 (Ym1), Mm00657889_mH; Arginase‐1 (Arg1), Mm00475988_m1; IL‐4Rα, Mm01275139_m1; TGFβ, Mm01178820_m1; IL‐10, Mm0‐0439614_m1; SOCS3, Mm01342740_g1; Hvcn1 (Hv1), Mm01199507_m1; GAPDH, Mm99999915_g1 (Applied Biosystems, Carlsbad, CA] on an QuantStudio™ 5 Real‐Time PCR System (Applied Biosystems). Samples were assayed in duplicate in 1 run (40 cycles), which was composed of three stages, 50°C for 2 min, 95°C for 10 s for each cycle (denaturation), and finally, the transcription step at 60°C for1 min. Gene expression was normalized by GAPDH and compared to the control sample to determine relative expression values by the 2−ΔΔCt method.

### 2.8 Western blotting

A tissue punch was taken from the perilesional area in ipsilateral cortex. Proteins from both perilesional cortex and ipsilateral hippocampus were extracted by RIPA buffer containing protease inhibitors and phosphatase inhibitor, then quantified by BCA assay. Twenty micro gram of proteins were loaded onto 4–20% SDS‐PAGE gels (Cat# 4561096, Bio‐Rad; Hercules, CA) as previously described (Sabirzhanov et al., 2019). Proteins were transferred onto nitrocellulose membranes and then blocked for 1 hr in 5% milk in 1× PBS containing 0.05% Tween 20 (PBS‐T) at room temperature. The membrane was incubated in rabbit anti‐HVCN1 (1:10000; Cat# AHC‐001, Alomone labs, Jerusalem, Israel), or mouse anti‐β‐actin (1:5000; Cat# A5441, Sigma‐Aldrich) overnight at 4°C, then washed 3 times in PBS‐T, and incubated in appropriate secondary antibodies for 2 hr at room temperature. Membranes were washed 3 times in PBS‐T, and proteins were visualized using SuperSignal West Dura Extended Duration Substrate (Cat# 37071, Thermo Scientific, Rockford, IL). Chemiluminescence was captured by ChemiDoc System (Bio‐Rad), and protein bands were quantified by densitometric analysis using Bio‐Rad Image Lab software. The data presented reflects the relative intensity of target protein band to that of the endogenous control β‐actin for each sample.

### 2.9 Beam walk

Fine motor function for each mouse was assessed with the beam walk test as described previously (Zhao, Sabirzhanov, Wu, Faden, & Stoica, 2015). For this experiment, mice were placed on one end of a wooden beam (5 mm width and 120 mm in length) suspended 200 mm in the air with support beams. The number of foot faults for the right hindlimb was recorded over 50 steps as the mouse traversed the beam. All mice were trained for three consecutive days before surgery and a basal level of performance was obtained immediately before surgery. Following surgery, the test was performed at 1, 3, 7, 14, 21, and 28 days after injury. All behavioral tests were performed by investigators blinded to the genotype or treatment groups.

### 2.10 Catwalk XT automated gait analysis

Motor coordination was performed and analyzed using the CatwalkXT automated system (Noldus; RRID:SCR_004074) (Ritzel et al., 2020). Each mouse underwent only one testing session after TBI to maintain situational novelty and encourage exploration of the CatWalk. Data acquisition took place in a darkened room with the same researcher handling each subject. The CatWalk itself features a red overhead lamp and green illuminated walkway, which responds to the pressure of the animals' weights and obtains live foot print videos. Each mouse was first placed in the open end of the CatWalk under the red ceiling light and allowed to walk across the walkway to the darkened escape enclosure. A minimum of three valid runs, or complete walkway crossings, were obtained for each subject. Trials in which the animal stopped partway across or turned around during a run were excluded from analysis.

### 2.11 Morris water maze

Spatial learning and memory were assessed between 37‐ and 41‐days postinjury with the Morris Water Maze as previously described in prior publications. This protocol consisted of two phases: (1) a hidden platform training phase for learning acquisition and (2) a probe test phase to assess reference memory. A circular tank (100 cm in diameter) was filled with water (23 +/− 2'C) and surrounded on four sides by various extra‐maze cues on the wall of the testing area. A transparent platform (10 cm in diameter) was submerged 0.5 cm below water surface within the northeast (NE) quadrant of the tank. Starting at 37 days postinjury, the mice were trained to find the submerged platform for four consecutive days (37–40 DPI). The mice underwent four trials in each day, starting from a randomly selected release point (east, south, west and north) and given a maximum of 90 s to find the submerged platform. If mice fail to find the platform within this time will be placed onto the platform and allowed to remain on the platform for 25 s on the first day of training and for 10 s on subsequent training days. For the probe test on day 41 postinjury, the platform was removed, and mice were released from the southwest (SW) position and the time spent in platform quadrant was recorded to assess retention of reference memory. The swim path, latency to platform, time spent in each zone and velocity was recorded with the Any‐Maze automated video tracking system.

Search strategy was assessed for each of the four trials on Day 4 of the training phase and the probe test as previously described (Zhao, Loane, Murray 2nd, Stoica, & Faden, 2012). Three strategies were identified with the using the following categorization scheme: (1) Spatial: swimming directly to platform with no more than one loop or swimming directly to the correct target quadrant and searching for the platform; (2) Systemic: searching for the interior portion of or the entire tank without spatial bias, and searching an incorrect target quadrant; (3) Looping: circular swimming around the tank, swimming in tight circles, and swimming around the wall of the tank. The percentage of each strategy in each group was calculated.

### 2.12 Y‐maze test

Y‐maze spontaneous alternation test was performed at 29 days postinjury to assess spatial working memory (Piao et al., 2013; J. Wu et al., 2016). The percentage of alternation is calculated on with the following equation: total alternations × 100/(total arm entries − 2). If a mouse scored significantly above 50% alternations (the chance level for choosing the unfamiliar arm), this was indicative of functional working memory.

### 2.13 | novel object recognition (NOR)

NOR was performed between 29‐ and 31‐days postinjury for a duration of three test days, which evaluated nonspatial hippocampal‐mediated memory as previously reported (Piao et al., 2013; J. Wu et al., 2016). Time spent with two identical objects was recorded; because mice inherently prefer to explore novel objects, a preference for the novel object (more time than chance [10 s] spent with the novel object) indicates intact memory for the familiar object.

### 2.14 | three chamber social interaction

Social interaction was performed at 72 days postinjury with the sociability and preference test originally designed by (Moy et al., 2004), using a three‐chambered rectangular apparatus made of Plexiglas with each chamber equally divided at 20 (width) × 40 (length) × 23 (height) cm. The open middle section allows free access to each chamber and two identical, wire cup‐like containers that are large enough to hold a single stimulus mouse were placed in the left and right‐side chambers, respectively. This test followed the same procedure described by earlier publications of our group (Ritzel et al., 2020). Briefly, this experiment consists of three 10 min phases with the test mouse starting off in the middle chamber for each phase. In the first phase, empty cages were placed the side chambers and the test mouse is allowed to freely explore all three chambers for a time period of 10 min in order to habituate to the novel environment. The second phase consists of a stimulus mouse and an object of interest randomly assigned to either the left or right chamber, during which the mouse is again allowed to explore for a duration of 10 min. In the third phase, the object of interest is exchanged for a second, unfamiliar stranger mouse. The contact time of the test mouse with each empty cup in the first phase, with the mouse and object in the second phase and with each mouse in the third phase are recorded as a test readout. The percentage between the amount of time spent with the novel stranger mouse and the familiar mouse in the third phase of the test indicates the sociability of each tested mouse.

### 2.15 lesion volume

Anesthetized mice using Euthasol were intracardially perfused with cold normal saline, followed by 4% paraformaldehyde. Whole brains were extracted minus the olfactory bulb and cut into coronal sections of 20 and 60 μm thickness on a cryostat (Leica), which were thaw‐mounted onto Superfrost Plus slides (Cat# 4951PLUS, Thermo‐Fisher). Lesion volume was performed on samples collected at 17 and 26 weeks after injury. Coronal sections cut at 60 μm thickness were stained with cresyl violet (Cat# PS102‐02, FD NeuroTechnologies, Baltimore MD). Quantification was based on the Cavalieri method using Stereoinvestigator software (MBF Biosciences; Zhao et al., 2015). The lesion volume was quantified by outlining the missing tissue on the injured hemisphere using Cavalieri estimator with a grid spacing of 0.1 mm. Every eighth section from a total of 96 sections was analyzed beginning from a random start point.

### 2.16 Neuronal loss

Quantified analysis of neuronal loss was carried out on samples collected at 26 weeks after injury and cut into 60 μm coronal sections. The optical fractionator method of unbiased stereology was used in the Stereoinvestigator software (MBF Biosciences, Williston, VT) to count the total number of surviving neurons in the ipsilateral cerebral cortex (Zhao et al., 2015). A total of six sections were analyzed for each animal and the total number of surviving neurons in each field was divided by volume of that region of interest to obtain an end result of counts/mm^3^, which reflects cellular density of neurons in the region.

### 2.17 | experimental design and statistical analyses

Data from individual experiments are presented as mean ± SEM, and individual data points are shown for each graph. Group effects were determined by two‐way ANOVA analysis with Tukey posthoc correction for multiple comparisons. For time course studies, differences between sham and each time point postinjury were analyzed by one‐way ANOVA using Dunnett's multiple comparison test. Behavioral data were also analyzed by two‐way repeated measures ANOVA (beam walk test) and Bonferroni's test or using Tukey's multiple comparison test to determine differences between sham and TBI. All behavioral, ex vivo, and molecular studies were performed by an investigator blinded to genotype and surgical condition. Statistical analysis was performed using the GraphPad Prism Software v. 6.0 (GraphPad Software, Inc., La Jolla, CA). *p* < .05 was considered statistically significant.

## 3 RESULTS

### 3.1 TBI induces intracellular microglial acidosis and extracellular brain acidosis in the first week postinjury

**FIGURE 2:**
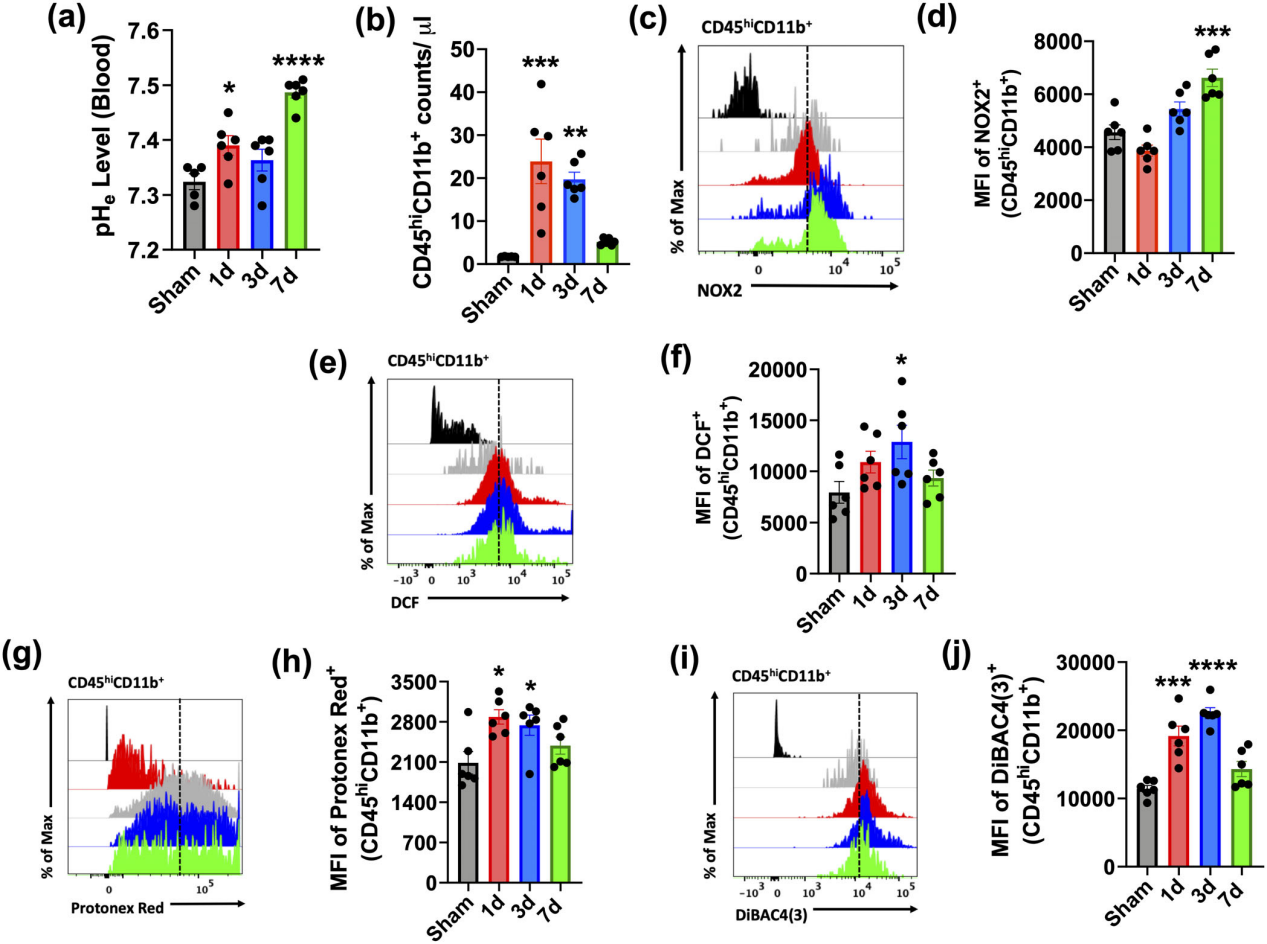
Intracellular changes in brain‐infiltrating myeloid cells during the first week of TBI. (a) Blood pH (pH_e_) was measured in sham mice and Day 1, 3, and 7 after TBI (*n* = 5‐6/group). Alkalosis was evident during the first week of injury. (b) Quantification of CD45^hi^CD11b^+^ infiltrating myeloid cell counts in the ipsilateral hemisphere during the first week of injury. (c) A representative histogram shows the relative mean fluorescence intensity of NOX2^+^ in the CD45^hi^CD11b^+^ population. (d) Mean fluorescence intensity of NOX2 expression was quantified. (e) A representative histogram shows the relative mean fluorescence intensity of DCF^+^ myeloid cells. (f) ROS production in infiltrating myeloid cells significantly peaked at day 3 after TBI relative to CD45^hi^CD11b^+^ cells present in the sham brain. (g) A representative histogram shows the relative mean fluorescence intensity of Protonex Red+ myeloid cells. (h) Intracellular pH was significantly lower in the CD45^hi^CD11b^+^ population at Day 1 and 3 after TBI compared to sham. (i) A representative histogram shows the relative mean fluorescence intensity of DiBAC4(3)^+^ myeloid cells. (j) Plasma membrane depolarization was significantly increased CD45^hi^CD11b^+^ cells at Day 1 and 3 after TBI compared to sham. For all flow cytometry experiments, *n* = 6/group. For all flow cytometry histograms, fluorescence minus one (FMO) controls are shown in black, time points are color coded according to bar graph, and a vertical fiducial line is included for reference. Max, maximum; MFI, mean fluorescence intensity. All data were analyzed by one‐way ANOVA using Dunnett's multiple comparison test to determine differences between sham and each time point postinjury (*p < .05; **p < .01; ***p < .001; and ****p < .0001) [Color figure can be viewed at wileyonlinelibrary.com]

Clinical studies on TBI patients show brain acidosis peaks at 24 hr after injury and gradually returns to physiological pH within the first week, mirroring the temporal profile of microglia proliferation and phagocyte activity (Clausen et al., 2005; Finnie, 2016). We first examined whether the CCI model of experimental TBI could replicate these features in mice. The extracellular pH of the perilesional area was measured by micro electrode (Figure 1a). We found that brain pH was significantly reduced at 24 hr, remaining at the same level for the first week after injury (Figure 1a,b; *p* < .0001). Local brain lactate concentrations (*p* < .01) and extracellular ROS levels (*p* < .0001) were also significantly increased during this time, albeit more modestly after 72 hr (Figure 1c,d; *p* < .05). To understand the relationship between brain acidosis and inflammation, we next assessed the functional dynamics of microglia activation. Using flow cytometry, we found that CD45^int^CD11b^+^ microglia proliferation was evident at 24 hr, with numbers remaining significantly higher than sham controls up to Day 7 after TBI (Figure 1e,f). Protein expression of NOX2 (Figure 1g,h) and cellular ROS production (Figure 1i,j, measured by H_2_DCFDA) peaked at 72 hr after TBI, with the latter remaining significantly higher at Day 7 relative to sham control (*p* = .020). Next, to determine whether activated microglia augment proton production and transport, we probed for changes in intracellular pH and plasma membrane potential, respectively. Cytosolic pH was dramatically lower in microglia after injury relative to sham, as evidenced by higher mean fluorescence intensity of the pH indicator Protonex red (Figure 1k‐l; *p* < .01). Increased membrane depolarization was also seen in microglia after TBI as shown by increased DiBAC4(3) fluorescence intensity, suggesting higher rates of proton extrusion across the membrane (Figure 1m,n). Interestingly, we observed a compensatory increase in blood pH levels after TBI (Figure 2a). However, similar changes in intracellular pH, ROS production, and membrane potential were also seen in the infiltrating myeloid (i.e., CD45^hi^CD11b^+^) population after injury (Figure 2b–j). Together, these data show that pH of microglia and brain is similarly decreased after brain trauma, demonstrating a strong association between microglial activation and brain acidosis following injury.

### 3.2 Elimination of microglia attenuates brain acidosis, oxidative stress, and neuroinflammation

**FIGURE 3:**
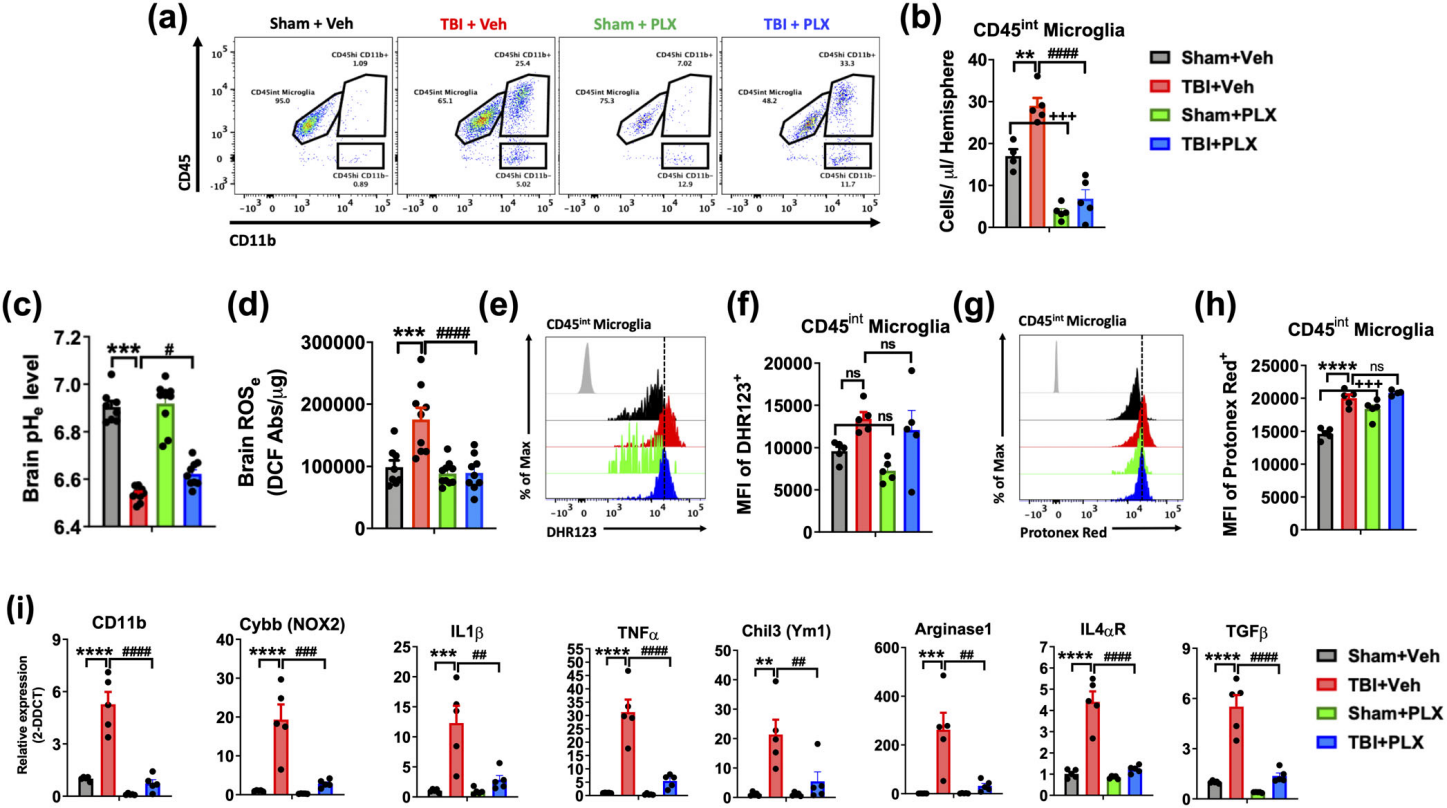
PLX5622‐mediated elimination of microglia attenuated brain acidosis, oxidative stress, and neuroinflammation. Mice were treated with vehicle or CSF1R antagonist PLX5622 chow for 2 weeks prior to surgery, until Day 3 postinjury. (a) A representative dot plot of brain leukocytes is shown. (b) Quantification of CD45^int^CD11b^+^Ly6C^−^ microglia counts in the ipsilateral hemisphere show PLX‐mediated elimination of microglia in sham and TBI‐treated groups. (c) Extracellular brain pH (pH_e_) was measured in sham vehicle, TBI vehicle, sham PLX, and TBI PLX mice (*n* = 8–10/group). (d) Extracellular reactive oxygen species (ROS_e_) levels were increased in vehicle‐treated mice after injury, but not PLX‐treated mice (*n* = 8–10/group). (e) A representative histogram shows the relative mean fluorescence intensity of DHR123^+^ microglia. (f) Increased microglial ROS production after TBI in vehicle and PLX‐treated mice. (g) A representative histogram shows the relative mean fluorescence intensity of Protonex Red^+^ microglia. (h) Cytosolic pH was decreased in sham PLX microglia compared to sham vehicle, and in TBI vehicle compared to sham vehicle. (i) Quantitative RT‐PCR analysis of gene expression for pro‐inflammatory (CD11b, NOX2, IL1β, and TNFα) and anti‐inflammatory (Ym1, Arginase 1, IL4αR, and TGFβ) mediators is shown (*n* = 5/group). Gene expression was normalized by GAPDH and expressed as a fold‐change relative to sham vehicle control. Data for (a–d) are representative of two independent experimental cohorts. For all flow cytometry experiments, *n* = 4‐5/group. For all flow cytometry histograms, fluorescence minus one (FMO) controls are shown in black, treatment and injury groups are color coded according to bar graph, and a vertical fiducial line is included for reference. Abs, absorbance, Max, maximum, MFI, mean fluorescence intensity, μg, microgram. All data were analyzed by two‐way ANOVA using Tukey's multiple comparison test to determine differences between sham and TBI (**p < .01, ***p < .001, and ****p < .0001) and vehicle and PLX groups (^#^ *p* < .05, ^##^ *p* < .01, ^###^ *p* < .001, and ^####^ *p* < .0001) [Color figure can be viewed at wileyonlinelibrary.com]

**FIGURE 4:**
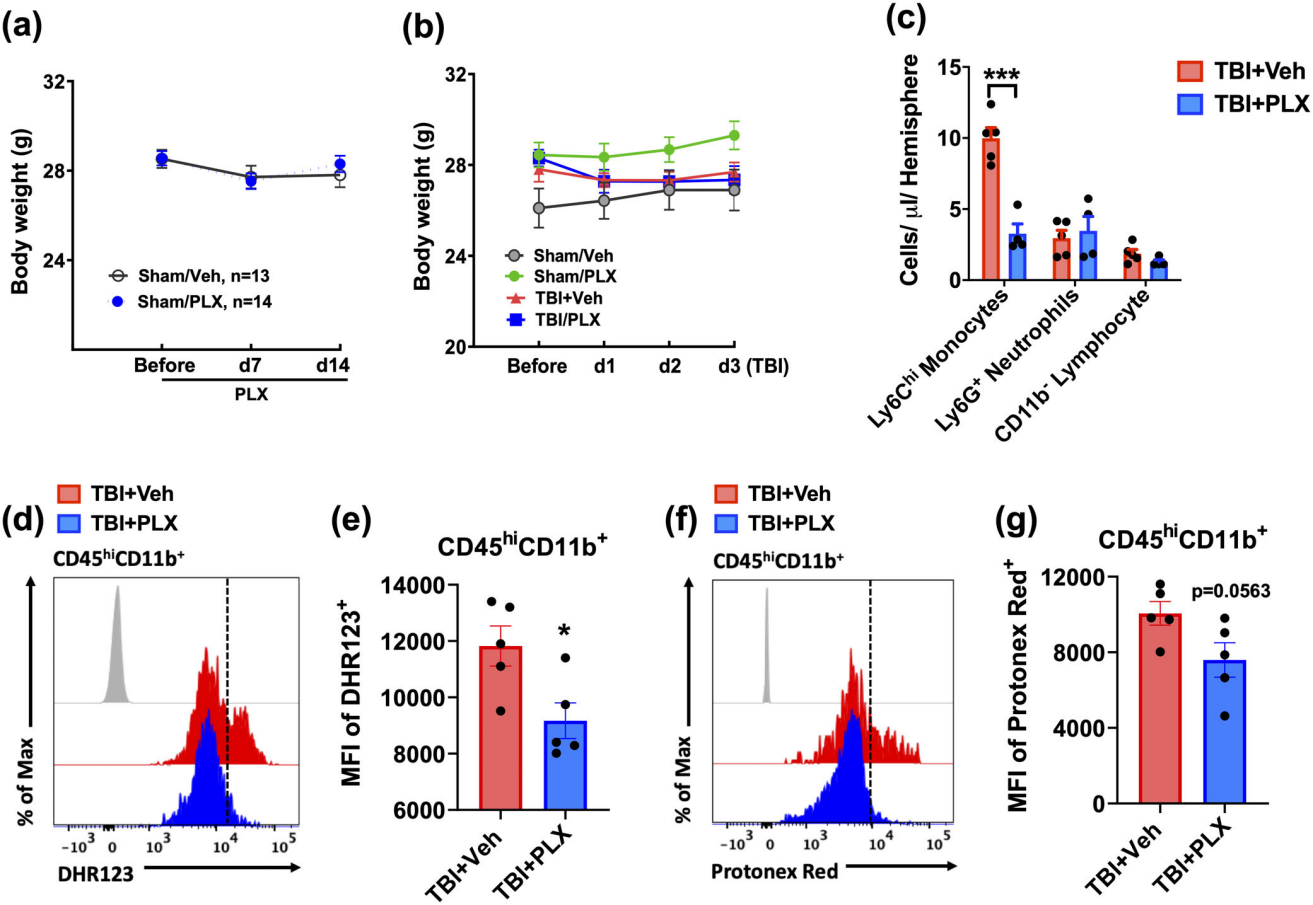
PLX5622‐mediated elimination of microglia attenuated brain infiltration of myeloid cells. Body weight was recorded for each treatment group prior to chow administration, at Day 7, and at 14 during treatment. (a) No change in body weight was seen between groups (*n* = 13–14/group). (b) No difference in body weight was found between TBI groups in the initial days after injury. (c) The number of infiltrating myeloid populations was quantified at 72 hr after TBI. PLX‐treated mice showed a dramatic reduction in Ly6C^hi^ monocyte infiltration compared to vehicle control after TBI. (d) A representative histogram shows the relative mean fluorescence intensity of DHR123^+^ myeloid cells. (e) ROS production in the infiltrating myeloid population was significantly lower in PLX‐treated mice compared to vehicle control. (f) A representative histogram shows the relative mean fluorescence intensity of Protonex Red^+^ myeloid cells. (g) The intracellular pH of CD45^hi^CD11b^+^ cells was significantly lower vehicle‐treated mice relative to the PLX‐treated group. For all flow cytometry experiments, *n* = 5/group. For all flow cytometry histograms, fluorescence minus one (FMO) controls are shown in black, treatment groups are color coded according to bar graph, and a vertical fiducial line is included for reference. μl, microliter; Max, maximum, MFI, mean fluorescence intensity. Data in (a,b) were analyzed by two‐way ANOVA with repeated measures. Data in (c) were analyzed by one‐way ANOVA using Sidak's multiple comparison test to determine differences cell count between treatment groups in each cell population postinjury (***p < .001). Data in (e,g) were analyzed using Student's *t*‐test (*p < .05) [Color figure can be viewed at wileyonlinelibrary.com]

The link between inflammation and acidosis implies that microglia could play an important role in modulating tissue pH levels after brain injury. To test this hypothesis, we performed microglia depletion experiments using the colony stimulating factor 1 receptor (CSF1R) antagonist, PLX5622. Neither PLX5622‐treatment nor TBI affected body weight (Figure 4a,b). Microglia numbers were dramatically lower (>80%) in PLX5622‐treated mice compared to control (Figure 3a,b, *p* < .0001). After 2 weeks on PLX5562 or vehicle chow, mice were subjected to CCI injury and examined for acidosis. The reduction in pH level of the perilesional area at 3 days postinjury was significantly attenuated in PLX5622‐treated versus vehicle‐treated mice (Figure 3c, *p* = .049). TBI‐induced increases in ROS level were also blunted after PLX5622 treatment (Figure 3d, *p* < .0001). Despite significantly fewer microglia in the PLX5622 group, cells in both groups exhibited intracellular acidosis and increased ROS production after injury as tested using two‐way ANOVA group analysis (Figure 3e–h, *F*_(1,16)_ = 63.28, *p* < .0001 and *F*_(1,16)_ = 11.71, *p* = .003, respectively). Tissue‐level expression of inflammatory markers confirmed that microglia depletion attenuated transcription of several important pro‐inflammatory genes (i.e., CD11b, NOX2, TNFα, and IL‐1β), and also key anti‐inflammatory genes (Ym1, Arg1, IL‐4Rα, and TGFβ; Figure 3i). In addition, a dramatic reduction in CD45^hi^CD11b^+^Ly6C^hi^ monocyte infiltration was found in PLX5562‐treated mice (Figure 4c). Within the broader CD45^hi^CD11b^+^ population, ROS production was significantly lower and cytosolic pH was relatively higher (Figure 4d–g). Together these findings suggest that microglia initiate and/or accelerate the acute inflammatory response to TBI and contribute to brain acidosis.

### 3.3 Brain acidosis persists for weeks after TBI and is associated with dysregulation of microglial Hv1 proton channel and NOX expression

**FIGURE 5:**
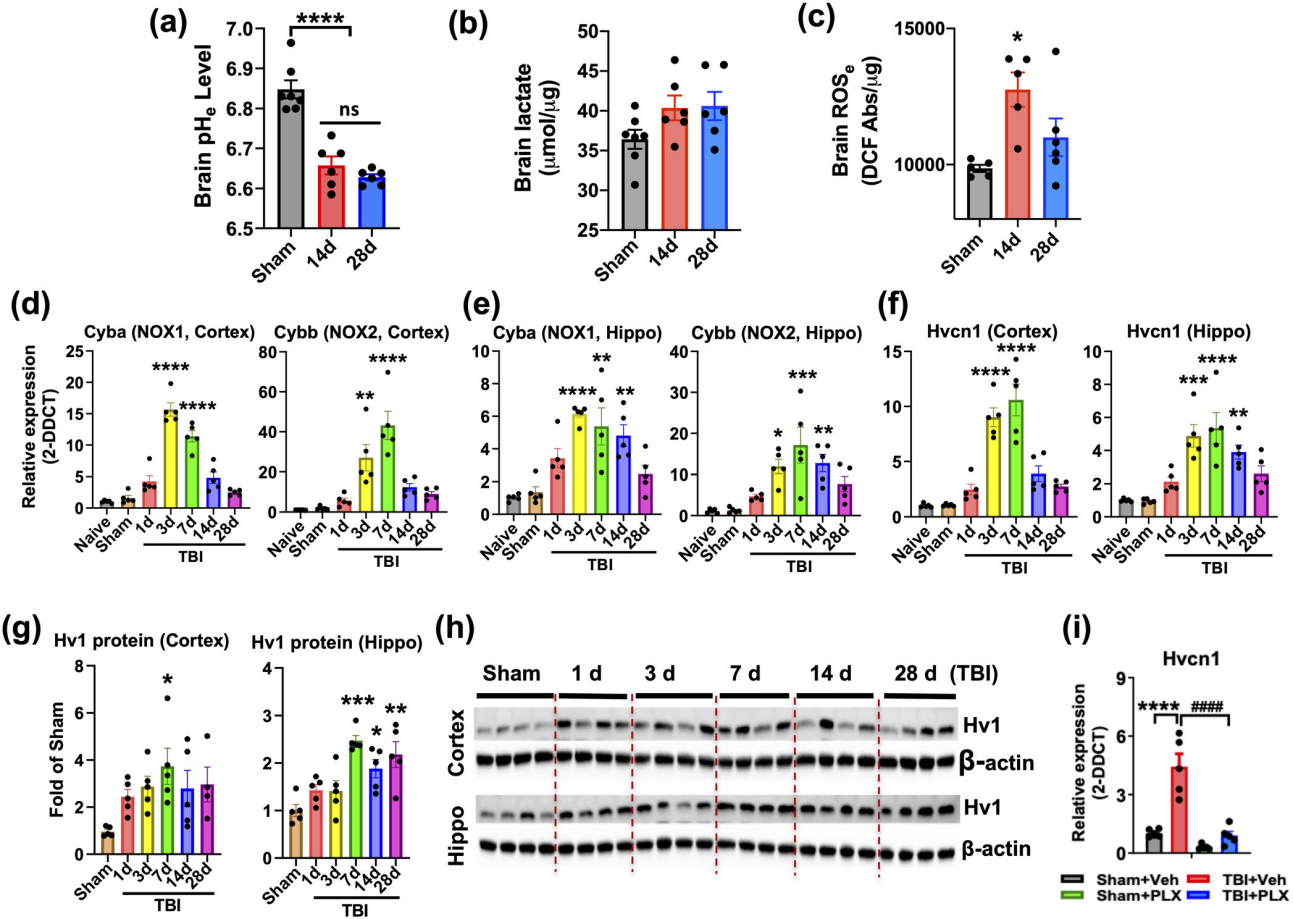
Brain acidosis persisted for weeks after TBI and was associated with dysregulation of microglial‐specific Hv1 proton channel and NOX expression. The long‐term duration of brain acidosis following TBI was examined. (a) Extracellular brain pH (pH_e_) was measured in sham mice and Day 14 and 28 after TBI (*n* = 7, 6, and 6/group). (b) Extracellular brain lactate concentrations are shown for each time point (*n* = 7, 6, and 6/group). (c) Extracellular reactive oxygen species (ROS_e_) levels in the cortex were increased relative to sham control (*n* = 5–6/group). Quantitative PCR analysis of gene expression of NOX1 and NOX2 in the cortex (d) and hippocampus (e) is shown. Gene expression of Hvcn1 (Hv1) was chronically upregulated in the cortex and hippocampus relative to sham control (f). Western blot analysis of protein expression for Hv1 in the cortex and hippocampus (g) and the representative blot image is shown (h). PLX5622‐treated mice showed a reduction in Hv1 gene expression relative to vehicle controls (i). Gene expression was normalized by GAPDH and expressed as a fold‐change relative to sham or vehicle control. Protein expression was normalized to β‐actin and expressed as a fold‐change relative to sham control. For all gene and protein expression experiments, *n* = 5/group. Abbreviations: Abs absorbance, hippo hippocampus, veh, vehicle, μmol, micromole, μg, microgram. Data for (a–g) were analyzed by one‐way ANOVA using Dunnett's multiple comparison test to determine differences between sham and each time point postinjury (*p < .05, **p < .01, ***p < .001, and ****p < .0001). Data for (i) was analyzed by two‐way ANOVA using Tukey's multiple comparison test to determine differences between sham and TBI (****p < .0001) and vehicle and PLX groups ^####^ *p* < .0001) [Color figure can be viewed at wileyonlinelibrary.com]

Because TBI is a chronic degenerative process, our finding that brain acidosis continued unabated during the first week after injury suggested that pH regulatory mechanisms could be impaired for a prolonged period. Extracellular pH measurements at Day 14 and 28 revealed that local acidosis in brain parenchymal tissue surrounding the lesion site persists more chronically in this moderate‐to‐severe TBI model (Figure 5a; *p* < .0001). But it should be noted that brain lactate concentrations were not significantly altered at these later time points (Figure 5b). Tissue ROS levels were however increased at Day 14 but not at Day 28 compared with the sham group (Figure 5c; *p* = .010). Gene expression of NOX1 and NOX2 enzymes in the cortex and hippocampus were acutely upregulated and statistically increased relative to sham controls up to 4 weeks after TBI as tested using one‐way ANOVA (Figure 5d,e). The relationship between NOX1/2, ROS, and pH led us to surmise that the proton channel Hv1 might be involved in TBI pathogenesis. Gene and protein expression of Hv1 in the cortex and hippocampus showed robust increases after TBI, which persisted for up to Day 28 (Figure 5f–h and Figure S2). Hv1 gene expression in the brain was virtually absent in PLX5622‐treated mice relative to vehicle controls, confirming earlier reports that this proton channel is primarily expressed in microglia (Figure 5i; *p* < .0001; L. J. Wu et al., 2012). These data indicate that brain acidosis following TBI is associated with perturbations in microglial Hv1 activity.

### 3.4 Hv1 promotes acid extrusion in microglia after TBI, exacerbating acidosis, oxidative stress, and neuroinflammation

**FIGURE 6:**
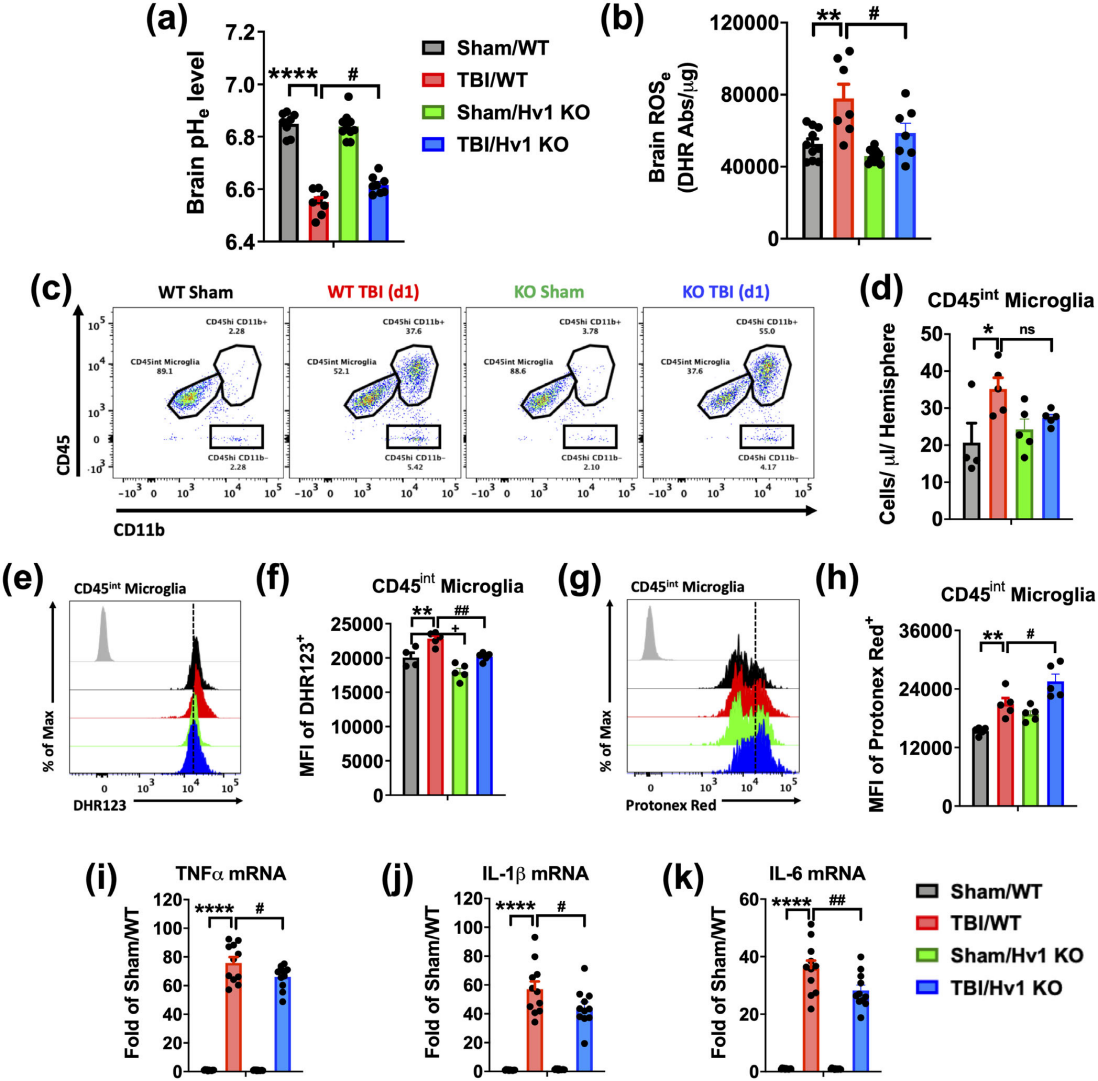
Hv1 augments acid extrusion in microglia after TBI, exacerbating acidosis, oxidative stress, and neuroinflammation. Acidosis and oxidative stress were examined in Hv1 KO mice and WT littermate controls at 24 hr after TBI. (a) Extracellular brain pH (pH_e_) was measured in sham/WT, TBI/WT, sham/Hv1 KO, and TBI/Hv1 KO mice (*n* = 8, 8, 10, and 8/group). (b) Extracellular reactive oxygen species (ROS_e_) levels were increased in WT mice after injury, but not KO mice (*n* = 7–10/group). (c) A representative dot plot of immune cells in the ipsilateral hemisphere is shown. (d) Quantification of CD45^int^CD11b^+^Ly6C^−^ microglia counts is shown. (e) A representative histogram shows the relative mean fluorescence intensity of DHR123^+^ microglia. (f) A significant group effect of genotype was seen in microglial ROS level, with KO mice displaying lower production in sham and TBI groups (*F*_(1,16)_ = 24.01, *p* = .0002). (g) A representative histogram shows the relative mean fluorescence intensity of Protonex Red^+^ microglia. (h) A significant group effect of genotype was seen in microglial pH, with KO mice displaying lower pH in sham and TBI groups (*F*_(1,16)_ = 15.40, *p* = .0012). Quantitative RT‐PCR analysis of gene expression for the pro‐inflammatory genes (i) TNFα, (j) IL1β, and (k) IL6 in the cortex show attenuated increases in KO mice after TBI relative to WT TBI (*n* = 11–12/group). Gene expression was normalized by GAPDH and expressed as a fold‐change relative to WT sham control. Data for (a,b, j–l) are representative of two independent experimental cohorts. For all flow cytometry experiments, *n* = 5/group. For all flow cytometry histograms, fluorescence minus one (FMO) controls are shown in black, genotype and injury groups are color coded according to bar graph, and a vertical fiducial line is included for reference. Abs, absorbance; KO, knockout; Max, maximum, MFI, mean fluorescence intensity; WT, wildtype; μg, microgram. All data were analyzed by two‐way ANOVA using Tukey's multiple comparison test to determine differences between sham and TBI (*p < .01, **p < .01, ***p < .001, and ****p < .0001) and WT and KO groups (^#^ *p* < .05, ^##^ *p* < .01, ^###^ *p* < .001, and ^####^ *p* < .0001) [Color figure can be viewed at wileyonlinelibrary.com]

Hv1 primarily functions to facilitate ROS production during oxidative burst and alleviate the subsequent intracellular acidosis via proton (i.e., H^+^) extrusion (DeCoursey, 2016). To determine whether Hv1 has a critical role in brain acidosis and neuroinflammation after TBI, we subjected Hv1 knockout (KO) mice and wildtype (WT) littermate controls to CCI and examined intracellular and extracellular changes in pH. Genetic ablation was confirmed at the gene and protein level (see Figure S3). At 24 hr postinjury, tissue acidosis was significantly decreased in Hv1 KO mice compared to WT (Figure 6a, *p* = .028). TBI‐induced increases in extracellular ROS level were also reduced in Hv1 KO mice (Figure 6b, *p* = .035). At the cellular level, microglia proliferation was significantly decreased in injured Hv1 KO mice compared to WT (Figure 6c,d). Moreover, we observed an overt decrease in ROS production and pH level in Hv1 KO microglia after injury relative to WT, consistent with notion that Hv1 is critical for both ROS generation and proton extrusion (Figure 6e–h, *p* = .006 and *p* = .027, respectively). Gene expression profiling in the cortex confirmed an attenuated increase in key pro‐inflammatory markers such as TNFα, IL‐1β, and IL‐6 in Hv1 KO mice relative to WT (Figure 6i–k).

**FIGURE 7:**
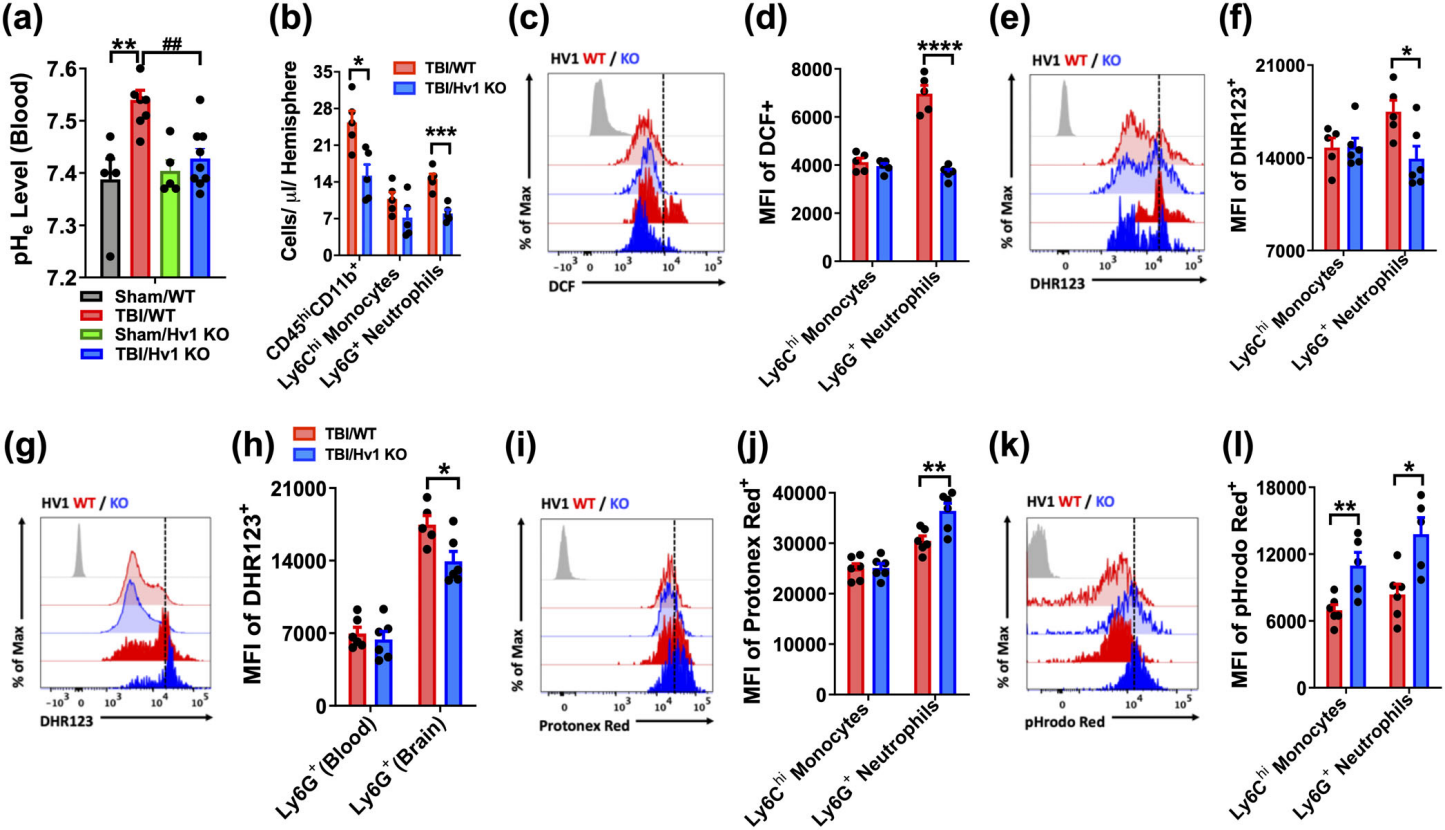
Intracellular acidosis and ROS production in brain‐infiltrating myeloid cells was altered in Hv1 KO mice at 24 hr after TBI. (a) Blood pH (pHe) was measured at 24 hr after TBI in WT and KO mice. Blood alkalosis was induced following TBI in WT mice but not KO mice (*n* = 5–9/group). (b) Quantification of CD45^hi^CD11b^+^ infiltrating myeloid populations in the ipsilateral hemisphere is shown. (c) A representative histogram of DCF^+^ infiltrating Ly6C^hi^ monocytes and Ly6G^+^ neutrophils shows the relative fluorescence intensity. (d) Quantification of DCF^+^ MFI shows a significant reduction in ROS production in neutrophils of Hv1 KO mice after TBI. (e) A representative histogram of DHR123^+^ infiltrating myeloid cells is shown. (f) Quantification of DHR123^+^ MFI shows a reduction in ROS production in Hv1 KO neutrophils consistent with (e). (g) A representative histogram of DHR123^+^ Ly6G^+^ neutrophils in the blood and brain at 24 hr postinjury is shown. (h) MFI quantification shows ROS production is significantly induced in neutrophils after they enter the injured brain. (i) A representative histogram of Protonex Red^+^ infiltrating myeloid cells shows the relative fluorescence intensity for WT and KO groups after TBI. (j) MFI quantification shows significantly lower cytosolic pH in KO neutrophils compared to WT control. (k) A representative histogram of pHrodo Red AM^+^ infiltrating myeloid cells is shown. (l) MFI quantification provides secondary confirmation that cytosolic pH is significantly lower in KO neutrophils compared to WT control. A significant difference between infiltrating monocyte groups was also seen. For all flow cytometry experiments, *n* = 5–6/group. For all flow cytometry histograms, fluorescence minus one (FMO) controls are shown in black, treatment groups are color coded according to bar graph, and a vertical fiducial line is included for reference. KO, knockout; Max, maximum; MFI, mean fluorescence intensity; WT wildtype; μl, microliter. Data in (a,d,f,h,j,l) were analyzed by two‐way ANOVA using Tukey's multiple comparison test to determine differences between sham and TBI (**p < .01) or WT and KO myeloid cells after TBI (*p < .05, **p < .01, and ****p < .0001), and between WT and KO TBI groups (##*p* < .01). Data in (b) were analyzed by one‐way ANOVA using Sidak's multiple comparison test to determine differences in cell count between genotype groups in each cell population postinjury (*p < .05 and ***p < .001) [Color figure can be viewed at wileyonlinelibrary.com]

**FIGURE 8:**
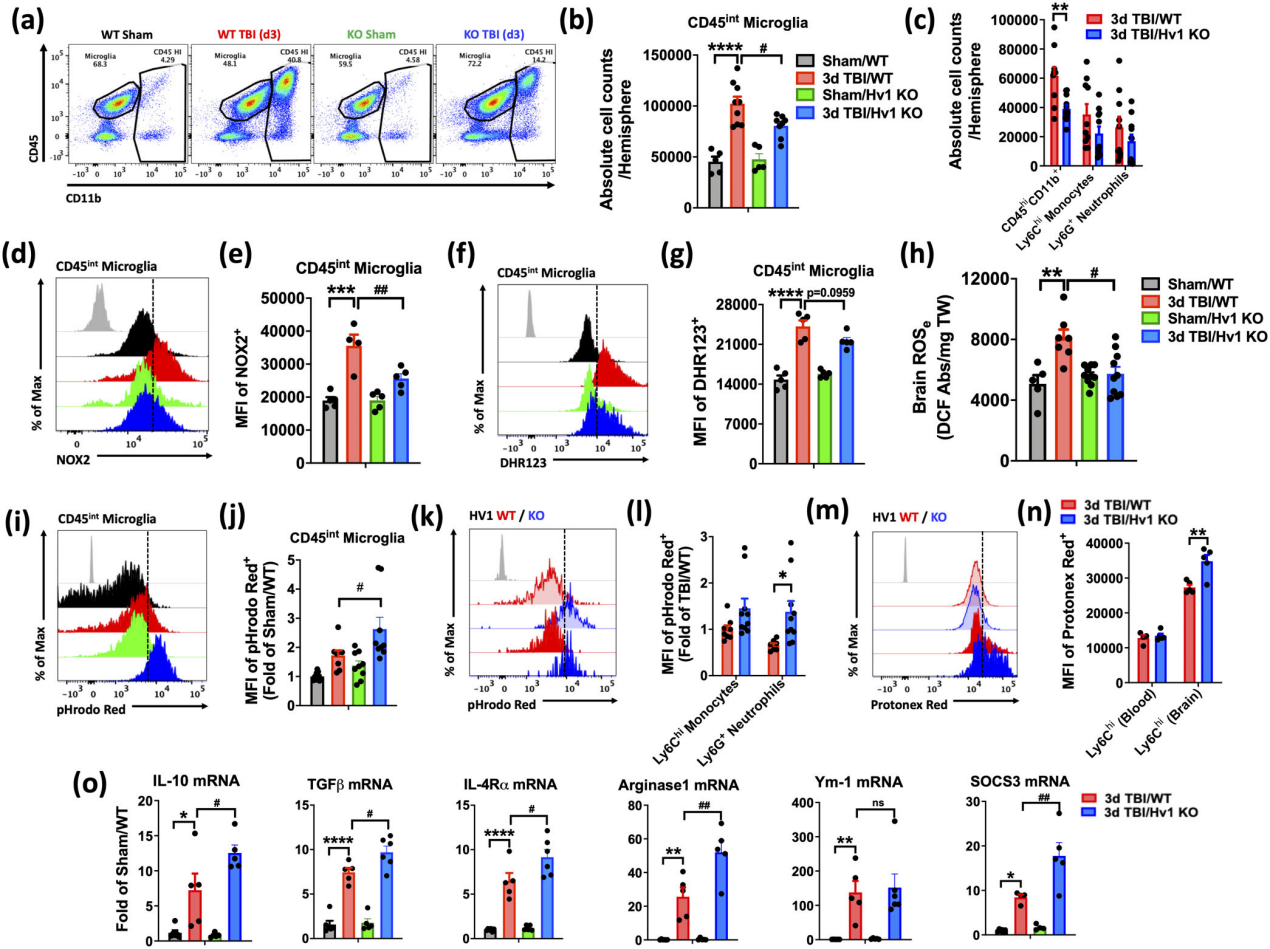
Neuroinflammation is attenuated in Hv1 KO mice at 72 hr after TBI. (a) A representative dot plot shows CD45^+^ immune cells in the brain of WT and KO, sham and TBI mice. (b) Total cell counts of CD45^int^CD11b^+^ microglia were significantly reduced in KO mice after injury compared to WT mice. (c) Total cell counts of brain‐infiltrating CD45^hi^CD11b^+^ populations is shown. (d) A representative histogram shows the relative mean fluorescence intensity of NOX2^+^ microglia. (e) Mean fluorescence intensity of NOX2 expression in microglia was quantified. (f) A representative histogram shows the relative mean fluorescence intensity of DHR123^+^ microglia. (g) MFI quantification of DHR123^+^ shows relative ROS production levels in microglia. (h) Extracellular brain ROS levels at 72 h after TBI are shown (*n* = 5–8/group). (i) A representative histogram shows the relative mean fluorescence intensity of pHrodo Red AM^+^ microglia. (j) MFI quantification was expressed as a fold‐change to WT sham control. (k) A representative histogram shows the relative mean fluorescence intensity of pHrodo Red AM^+^ of brain‐infiltrating monocytes and neutrophils. (l) MFI quantification was expressed as a fold‐change to the WT monocyte group, and a significant decrease in intracellular pH was seen in KO neutrophils compared to WT control. (m) A representative histogram of Protonex Red^+^ monocytes in the blood and brain at 72 hr postinjury is shown. (n) MFI quantification shows intracellular pH is significantly reduced in monocytes after they enter the injured brain. (o) Quantitative RT‐PCR analysis of gene expression for the anti‐inflammatory genes: IL10, TGFβ, IL4Rα, Arginase 1, Ym1, and SOCS3 in the cortex show significantly greater increases in KO mice after TBI relative to WT TBI (*n* = 4–6/group). Gene expression was normalized by GAPDH and expressed as a fold‐change relative to WT sham control. Data for (b,c,j,l) are representative of two independent experimental cohorts (*n* = 4–5/group). For all other flow cytometry experiments, *n* = 5/group. For all flow cytometry histograms, fluorescence minus one (FMO) controls are shown in black, genotype and injury groups are color coded according to bar graph, and a vertical fiducial line is included for reference. Abs, absorbance; KO, knockout; Max, maximum, MFI, mean fluorescence intensity; mg, microgram; ns, not significant; WT, wildtype. All data were analyzed by two‐way ANOVA using (c,l,n) Sidak's or (b,e,g,h,j,o) Tukey's multiple comparison test to determine differences between sham and TBI (*p < .01, **p < .01, ***p < .001, and ****p < .0001) and WT and KO groups (^#^ *p* < .05 and ^##^ *p* < .01) [Color figure can be viewed at wileyonlinelibrary.com]

TBI‐induced blood alkalosis was attenuated in Hv1 KO mice (Figure 7a). Examination of brain‐infiltrating leukocytes revealed a dramatic reduction in Ly6G^+^ neutrophils in KO mice compared to WT control (Figure 6c and Figure 7b). Reductions in neutrophil‐produced ROS in KO mice were evident after entry into the injured brain, suggesting impairment in oxidative burst (Figure 7c–h). Consistent with our earlier results, brain‐infiltrating neutrophils from KO mice exhibited significantly greater intracellular acidosis (Figure 7i–l). Similar changes were also seen in KO mice at 72 hr postinjury, including reductions in microglia and infiltrating leukocyte counts (Figure 8a–c), attenuated oxidative burst activity and cortical tissue ROS levels (Figure 8d–h), and increased intracellular acidosis (Figure 8i,j). Despite being fewer in number, infiltrating myeloid cells in KO mice also exhibited lower pH levels relative to WT control after injury (Figure 8k,l, *F*_(1,31)_ = 9.711, *p* = .003). These changes were seen in the brain, but not blood, indicating that the injury environment alters pH regulation of other phagocytes as well (Figure 8m,n, *p* = .001). At 72 hr, KO mice displayed higher brain expression of anti‐inflammatory cytokines and signaling molecules, suggesting earlier resolution of neuroinflammation compared to injured WT mice (Figure 8o). Taken together, these findings suggest that oxidative burst activity in microglia contribute to brain acidosis and TBI pathology via Hv1‐mediated proton extrusion into the extracellular space as well as proinflammatory mediators.

### 3.5 Depletion of Hv1 limits neurodegeneration after TBI and improves functional outcome

**FIGURE 9:**
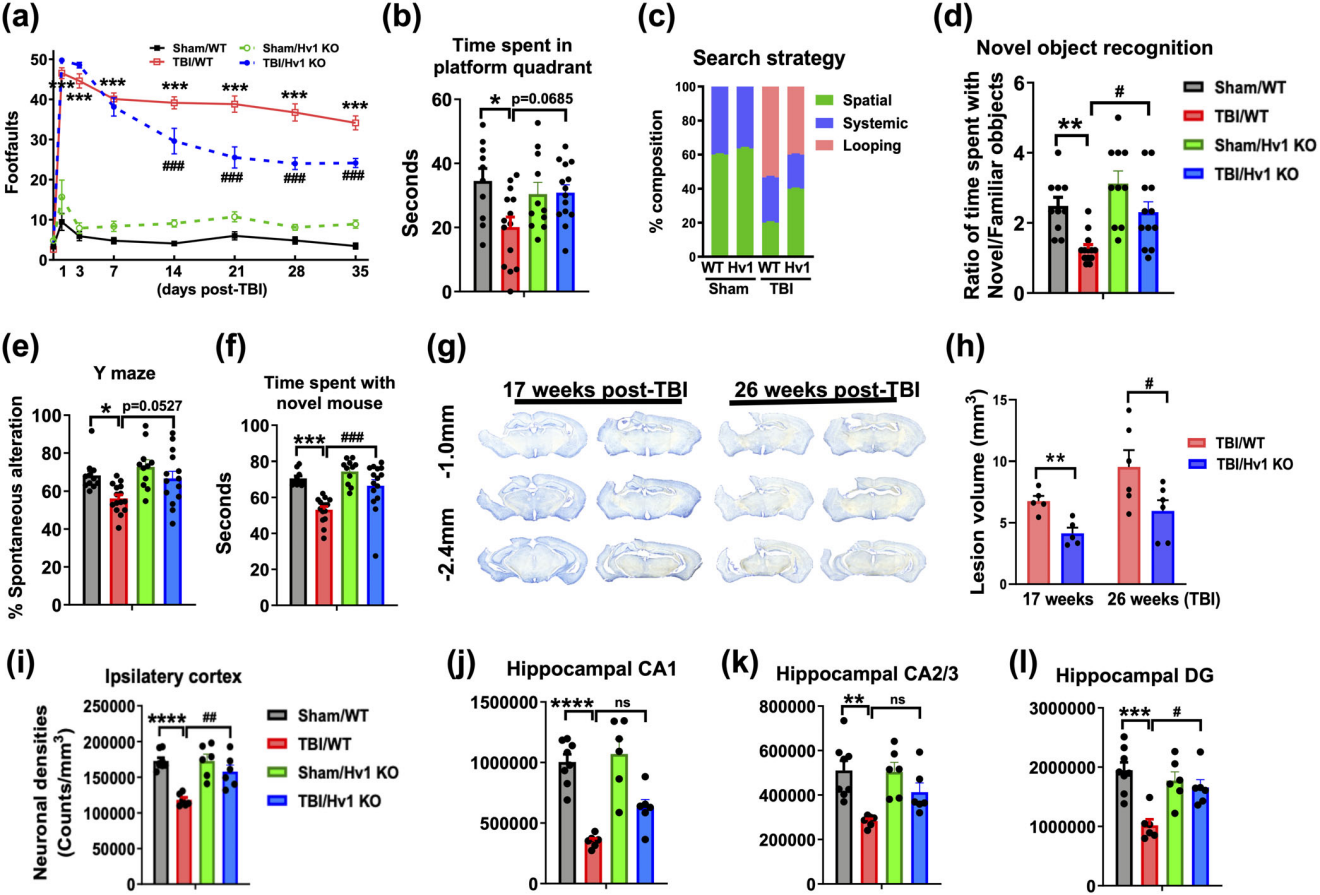
Hv1 KO mice exhibit lasting neuroprotection after TBI and improved functional recovery. Long‐term neurological outcomes of Hv1 KO mice were assessed. (a) Genetic ablation of Hv1 significantly enhanced long‐term fine motor function at 14, 21, 28, and 35 days postinjury as shown by reduced number of footfalls in a beam walk test. (b) At 5 weeks postinjury, TBI significantly decreased time spent in the platform quadrant in WT but not KO mice during the Morris water maze task. (c) The frequency of each escape strategy (spatial, systematic, and looping) used in each group is shown. KO TBI mice spent a higher percentage of time using spatial strategy and less time using the looping strategy compared to WT TBI. (d) At 4 weeks postinjury, TBI significantly decreased time spent with the novel object versus familiar object in a novel object recognition task, however, KO TBI animals spent comparably more time with novel objects than WT TBI. (e) At 4 weeks postinjury, TBI decreased the percentage of spontaneous alterations in the Y‐maze test, when compared to sham counterparts. (f) Social interaction was performed at 72 days postinjury. TBI reduced the contact time spent with a novel “stranger” mouse following removal of a familiar object. (g) Representative coronal sections of cresyl violet‐stained brains depict the lesion volume in each TBI group at 17 and 26 weeks postinjury. (h) Lesion volume was ~2‐fold greater in WT TBI compared to KO TBI (*n* = 5–6/group). Stereological cell counting of histological sections showed that TBI significantly decreased the number of Nissl‐stained neurons/mm^3^ in the (i) ipsilateral cortex and (j) CA1, (k) CA2/3, and (l) DG regions of the ipsilateral hippocampus (*n* = 6–8/group). or all behavioral experiments, *n* = 10–15/group. CA, cornu ammonis; DG, dentate gyrus; KO, knockout; mm millimeter, WT, wildtype. All data were analyzed by two‐way ANOVA with repeated measures (beam walk test) and Bonferroni's test or using Tukey's multiple comparison test to determine differences between sham and TBI (*p < .01, **p < .01, ***p < .001, and ****p < .0001) and WT and KO groups (^#^ *p* < .05, ^##^ *p* < .01, and ^###^ *p* < .001) [Color figure can be viewed at wileyonlinelibrary.com]

Clinical studies have shown that brain acidosis severity is a strong predictor of long‐term functional outcome (Clausen et al., 2005; Ellingson et al., 2019). To that end, we performed a battery of behavioral tests and histopathological examination to determine the chronic impact of acute brain acidosis on recovery (see Figure S4). Hv1 KO mice exhibited marked motor improvement in the beam walk test beginning at day 14 after TBI, in contrast to WT mice (Figure 9a, *p* < .0001). Results from Morris water maze, NOR, and *y*‐maze task showed significantly better spatial learning and recognition memory in Hv1 KO mice following TBI (Figure 9b–e). No difference between genotype in swim speed was recorded in sham or injury for the Morris water maze (Figure S4). Social interaction testing at 10 weeks postinjury showed that Hv1 KO mice spent significantly more time interacting with a novel mouse compared to WT (Figure 9f, *p* < .0001). No changes in gait dynamics were observed between groups (data not shown). Cresyl violet staining revealed significantly smaller lesion volumes in Hv1 KO mice at 17 and 26 weeks after TBI (Figure 9g,h, *p* = .003 and *p* = .048, respectively). Closer examination using stereological quantification found that Hv1 KO mice had greater preservation of neuron densities in the ipsilateral cortex and dentate gyrus region of the hippocampus (Figure 9i–l, *p* = .003 and *p* = .015, respectively). Together these findings demonstrate that attenuating brain acidosis in Hv1 deficiency in the immediate aftermath of TBI is associated with less neuronal degeneration and improved functional recovery during the chronic stages of the disease.

## 4 DISCUSSION

In this study, we investigated the cellular and molecular mechanisms of brain acidosis after head injury. Decreased extracellular (i.e., perilesional cortex) and intracellular (e.g., microglia) pH were demonstrated in a well‐characterized, rodent TBI model, with acidosis persisting for weeks. Depletion experiments revealed a critical role for microglia in regulating tissue acidosis, oxidative stress, and neuroinflammation during the acute stages of injury. Working in concert with NOX, the Hv1 proton channel functions as an acid extrusion mechanism required for compensating cytosolic pH changes in activated microglia. Hv1 expression was chronically upregulated in the brain after head injury. As demonstrated in Hv1‐deficient mice, attenuating brain acidosis in the acute period after TBI limited subsequent neurodegeneration and long‐term neurological impairment.

Most of our knowledge of TBI acidosis is derived from clinical studies, so our understanding of this secondary injury mechanism is limited. More than three decades ago, our laboratory was among the first to show acute pH alterations in the injured rat brain using proton (1H) magnetic resonance spectroscopy (McIntosh, Faden, Bendall, & Vink, 1987). Since then, to our knowledge only one experimental study has examined brain pH in situ after TBI (Yin et al., 2013). The authors similarly used a pH meter probe to demonstrate acidosis in brain tissue after fluid percussion injury. However, pH was only measured at a single early time point (60 min postinjury) in mice receiving mechanical ventilation under anesthesia. Our study included temporal profiling of extracellular and intracellular pH. We show that microglial acidification occurs in conjunction with brain acidification, and that depleting microglia attenuates brain acidosis. PLX5622‐mediated depletion of microglia markedly reduced extracellular ROS levels and biomarkers of inflammation, including leukocyte infiltration. These findings are consistent with a recent study showing that microglial depletion promoted neurite outgrowth, preserved dendritic spines, and reduced neuronal apoptosis and decreased infiltrating myeloid cells at 24 and 72 hr after fluid percussion injury (C. F. Wang et al., 2020). Because ROS activity and acidosis were a prominent feature in microglia as well as at the lesion site, it suggested that NAPDH oxidase coupled Hv1 activity might play a role in chronic neuroinflammation after TBI. We and others have recently reported that delayed elimination and repopulation of microglia limited TBI‐associated neuropathological changes at 3 months postinjury (Henry et al., 2020; Willis et al., 2020). PLX‐treated mice showed reductions in lesion volume, decreased NOX2 expression, and enhanced functional recovery. Although that study did not assess changes in brain pH, it is plausible that chronic microglial activation, by causing constitutive release of ROS/H^+^, can promote neuronal degeneration in part through long‐term acidosis‐mediated neurotoxicity.

Our study is the first to demonstrate long‐term brain acidosis after head injury. Although our data support the novel hypothesis that microglia have an important role in regulating the severity of brain acidosis and long‐term functional outcomes after TBI through a proton extrusion mechanism, coincident lactic acidosis may be seen as a potential confounder to this interpretation. In WT mice, TBI caused a local increase in lactate concentration that peaked at 24 hr and remained elevated until Day 7. Importantly, at chronic posttraumatic timepoints there were no changes in lactate concentrations, yet extracellular pH remained consistently below sham levels. This suggests that whereas lactate accumulation is a fundamental consequence of hyperglycolytic activity early after injury, other pH regulatory mechanisms drive or maintain the acidification of the injury environment late after TBI. Indeed, we found that Hv1 expression is chronically upregulated for weeks after TBI and functions as a critical modulator of disease progression. Whether brain acidosis is a chronic feature of posttraumatic brain injury in humans warrants further investigation.

Although the Hv1‐driven reductions in brain pH are modest, it is important to emphasize that a pH decrease from 7.0 to 6.5, as found in our study, reflects a fivefold increase in the concentration of hydrogen ions after injury. Due to the logarithmic scale, small changes in tissue pH level can have considerable biological consequences. DeCoursey and Hosler (2014) reported that activation of a single Hv1 channel can allow up to 100,000 hydrogen ions across the membrane each second (DeCoursey & Hosler, 2014). The fate of those ions after they reach the extracellular space is not completely understood. Our previous study showed that Hv1 proton extrusion could directly activate acid‐sensing ion channel 1a (ASIC1a) and mediate neuronal pathophysiology (Zeng et al., 2015). Nevertheless, several groups have shown that mild acidosis may be neuroprotective, implying that acid–base sensing and communication between cells is essential for restoring cellular homeostasis after an insult. Early work by Simon and colleagues demonstrated that hypercarbic‐induced brain acidosis reduced stroke infarct in rats in a biphasic manner (Simon, Niro, & Gwinn, 1993). Maximal protection was observed at pH 6.8, but the effect was lost at pH 6.5. Transient mild acidosis treatment (i.e., acidic postconditioning) has been demonstrated to provide neuroprotection in vivo, comparable to ischemic postconditioning, resulting in significantly smaller infarct volumes in mice at 72 hr poststroke (Fan et al., 2014; Shen et al., 2017; Zheng et al., 2017). Importantly, the authors noted that although relatively mild pH unit decreases of ~0.16–0.24 in the ischemic cortex were associated with robust neuroprotection, decreases below ~0.36 failed to show any protection, and hence, exhibited more brain damage (Fan et al., 2014). Thus, it is critical to distinguish between adaptive or compensatory decreases in pH and those occurring in association with neurodegeneration. This suggests the term *pathological* acidosis refer to decreases in brain pH that fall below a certain buffering threshold (~6.6), at which activated endogenous neuroprotective pathways are negated by acidotoxicity.

The neuroprotective effects of Hv1 deficiency have been observed in multiple models of CNS injury and disease. Although we are the first to demonstrate these effects in experimental TBI, others have previously identified a role for Hv1 in modulating neuroinflammation in other model systems. For example, Hv1 KO mice exhibited less glial activation, decreased production of pro‐inflammatory cytokines, and an M2‐dominant polarization phenotype in models of ischemic stroke, chronic hypoperfusion, multiple sclerosis, and spinal cord injury (Li et al., 2020; Liu et al., 2015; L. J. Wu et al., 2012; Yu et al., 2020). In each case, the inflammatory profile was associated with cellular protection. Wu and colleagues were the first to demonstrate that Hv1 mediated oxidative damage in the ischemic brain using histological staining with dihydroergotamine (DHE) (L. J. Wu et al., 2012). Indeed, it is now evident that Hv1 is essential for ROS formation during oxidative burst activity in phagocytes such as microglia. However, Hv1 primarily functions as an acid extruder, channeling protons into the phagosome or across the plasma membrane (Ramsey et al., 2009). No studies to date have examined how this major aspect of Hv1 function is perturbed after brain injury. Our data suggest that TBI causes a simultaneous increase in microglial acidification and ROS production, coincident with tissue acidification and oxidative stress. Others have previously shown that activated microglia can acidify the extracellular milieu (L. Wang et al., 2019). While infiltrating myeloid cells may also contribute to early brain acidosis, total numbers pale in comparison to microglia, return to sham brain levels by 7 days, and are significantly reduced in Hv1 KO and PLX5622‐treated groups, suggesting microglia are the predominant drivers of Hv1‐mediated acidosis and chronic TBI pathology. Furthermore, RNA silencing of Hv1 prevented extracellular acidification, providing direct evidence for proton extrusion across the plasma membrane (Y. Wang, Li, Wu, Che, & Li, 2012). Although it was known that oxidative burst activity in microglia increases Hv1 activity to compensate the charge created by electrogenic NOX activity, the potential hazards of this process (i.e., extracellular acidosis) and its pathological consequences following head injury are rarely discussed. Although the present study highlights the importance of proton transport regulation in brain injury‐induced acidosis, future studies are required to understand how an excess of hydrogen ions directly impacts neighboring cells. It was previously postulated that microglia may communicate with neurons via Hv1‐mediated release of H^+^ and ROS (L. J. Wu, 2014; Zeng et al., 2015). Indeed, voltage‐insensitive proton‐gated cation channels known as ASICs are expressed on neurons and can be activated by low pH. Moreover, ASIC1a‐deficient mice show reduced neurodegeneration after focal ischemia and experimental TBI (Y. Z. Wang et al., 2015; Yin et al., 2013). It remains to be seen whether targeting Hv1 in the chronic phase of injury could be a viable delayed treatment approach. Small molecule inhibitors of the Hv1 channel have been shown to act as chemotherapeutics and anti‐inflammatory agents (Hong, Pathak, Kim, Ta, & Tombola, 2013). However, the specificity of Hv1 inhibitors has been debated. Future studies are needed to determine whether selective inhibition of the Hv1 channel can reduce brain acidosis and improve neurological outcome after head injury.

One major discrepancy between our results and the existing clinical data is that while human brain pH abruptly decreased in the first 6 hr to ~6.85, surviving TBI patients with poor outcomes ultimately restored pH balance back to ~7.05–7.20 by 24 hr (Clausen et al., 2005). Although there are caveats regarding injury heterogeneity, severity, and the location of pH measurements in human subjects that can be better controlled for in experimental settings, the measurements made in our study were performed on freshly perfused intact brains ex vivo, rather than in living animals with continuous blood flow circulation. The pH of blood is significantly higher than brain parenchymal tissue, and significantly increases after TBI (see Figure 2a). Thus, pH measurements in the living brain, which is highly vascularized, may be further confounded by trauma‐induced hemorrhage and mask any acidosis in the brain parenchyma. Indeed, our unpublished results show that brain acidosis is virtually absent when measured ex vivo in unperfused brain tissue. Moreover, placement of the pH probe in the epicenter of the injury (i.e., impact site) where hemorrhage is most pronounced during the first several days after CCI can also result in higher pH readouts. To this end, brain pH measurements in living animals may be dependent on changes in systemic circulatory pH‐regulating factors. Although paradoxical, our findings suggest that pH measurements in brains perfused with ice‐cold saline, collected within minutes of dissection and recorded around the perilesional cortex may provide a more accurate measure of parenchymal tissue pH levels.

While our study defines a role for Hv1 in TBI and directly implicates microglia as a novel cellular mediator linking inflammation to acidosis, we acknowledge several limitations to this work. Despite the fact that men are at greater risk for concussion due to greater participation in high‐risk activities, women tend to report more symptoms and more persistent sequelae following concussion (Rubin & Lipton, 2019). Our group has recently shown that female mice exhibit protection during the acute stages of CCI injury (Doran et al., 2019). Of note, we reported sex differences in neuroinflammation and behavioral deficits. Male mice were included in the present study to reduce the number of animals used and reduce confusion across the various experimental paradigms of the study (e.g., time course dynamics, PLX‐treatment groups, genotype groups, etc.). Hv1‐related sex differences in brain pathology have not been reported and remain an intriguing area for future investigation. Additionally, although decreased acidosis was associated with better outcomes in Hv1 KO mice, its modest impact on brain pH make it clear that that the Hv1 proton channel represents just one of the several known acidosis‐related secondary injury mechanisms, including the ubiquitously expressed Na^+^/H^+^ exchangers (NHEs). Indeed, the data appear to support the notion that tissue acidosis is primarily driven by nonmicroglial CNS cells. Astrocytes are also reported to be chronically activated after TBI, however our study did not address these changes. Extracellular acidosis has been demonstrated to have differential effects on glial function, with astrocytes exhibiting higher sensitivity to pH‐mediated deficits in Aβ clearance (Eugenin et al., 2016). The interplay between glial cells during acidosis requires further study. Lastly, it is important to note that expression of CSF1R, the primary target of PLX5622, can be detected on other myeloid cells such as peripheral tissue resident macrophages, dendritic cells, and myeloid‐derived suppressor cells. Thus, CSF1R inhibitors likely have off‐target effects in various peripheral tissues including skin, heart, liver, small intestine, and lung (Lei et al., 2020). Therefore, cautious interpretation of microglia‐specific depletion effects using systemic delivery and currently available small‐molecule CSF1R inhibitors is warranted.

In conclusion, we report that microglia contribute to pathological acidosis after TBI. Our results establish a direct connection between acidosis and inflammation. Mechanistically, we determined that the voltage‐gated proton channel Hv1 is a key driver of tissue acidosis, oxidative stress, and neuroinflammation. We demonstrate that TBI causes intracellular and extracellular acidosis that persist chronically. Therefore, it may be possible to reverse the effects of pathological acidosis and improve recovery by targeting Hv1 proton channel, even in the chronic stages of injury.

## CONFLICT OF INTEREST

The authors declare no conflict of interest.

## AUTHOR CONTRIBUTIONS

Rodney M. Ritzel contributed to study conception and design, performed the extracellular brain pH measurements, the extracellular brain ROS assay, designed, performed, and analyzed the flow cytometry experiments, wrote the manuscript and prepared figures; Junyun He performed mice CCI surgeries, the qPCR and WB assay and quantification, assisted with the flow cytometry experiments, collected data, and manuscript preparation; Yun Li performed behavioral tests, the extracellular brain pH measurements and lactate assay, and data analysis, assisted with the flow cytometry experiments, collected data, and manuscript preparation; Tuoxin Cao performed neuronal counts using unbiased stereology; Niaz Khan largely assisted with the acidosis flow cytometry experiments; Bosung Shim performed lesion volume analysis; Bosung Shim performed Hv1 PCR analysis; Taryn Aubrecht contributed mice surgeries; Bogdan A. Stoica contributed with experimental design; Alan I. Faden contributed to discussion and manuscript preparation; Long‐Jun Wu provided the Hv1 KO mice, project discussion, and manuscript revision. Junfang Wu contributed to study conception and design, behavioral data analysis, acquiring the PLX5622/vehicle chow, wrote the manuscript and prepared figures. All authors read and approved the manuscript prior to submission.

## Supporting information


**Appendix** S1: Supporting information

## Data Availability

The data that support the findings of this study are available from the corresponding author upon reasonable request.
