# Supplementary material for: Proton extrusion during oxidative burst in microglia exacerbates pathological acidosis following traumatic brain injury
Source: Glia. 2020 Oct 22;69(3):746–64. doi: 10.1002/glia.23926 (PMC7819364; doi:10.1002/glia.23926)
Supplement: Supplementary file 1 — Appendix S1: Supporting information [file GLIA-69-746-s001.docx]

**Supporting Information**

**
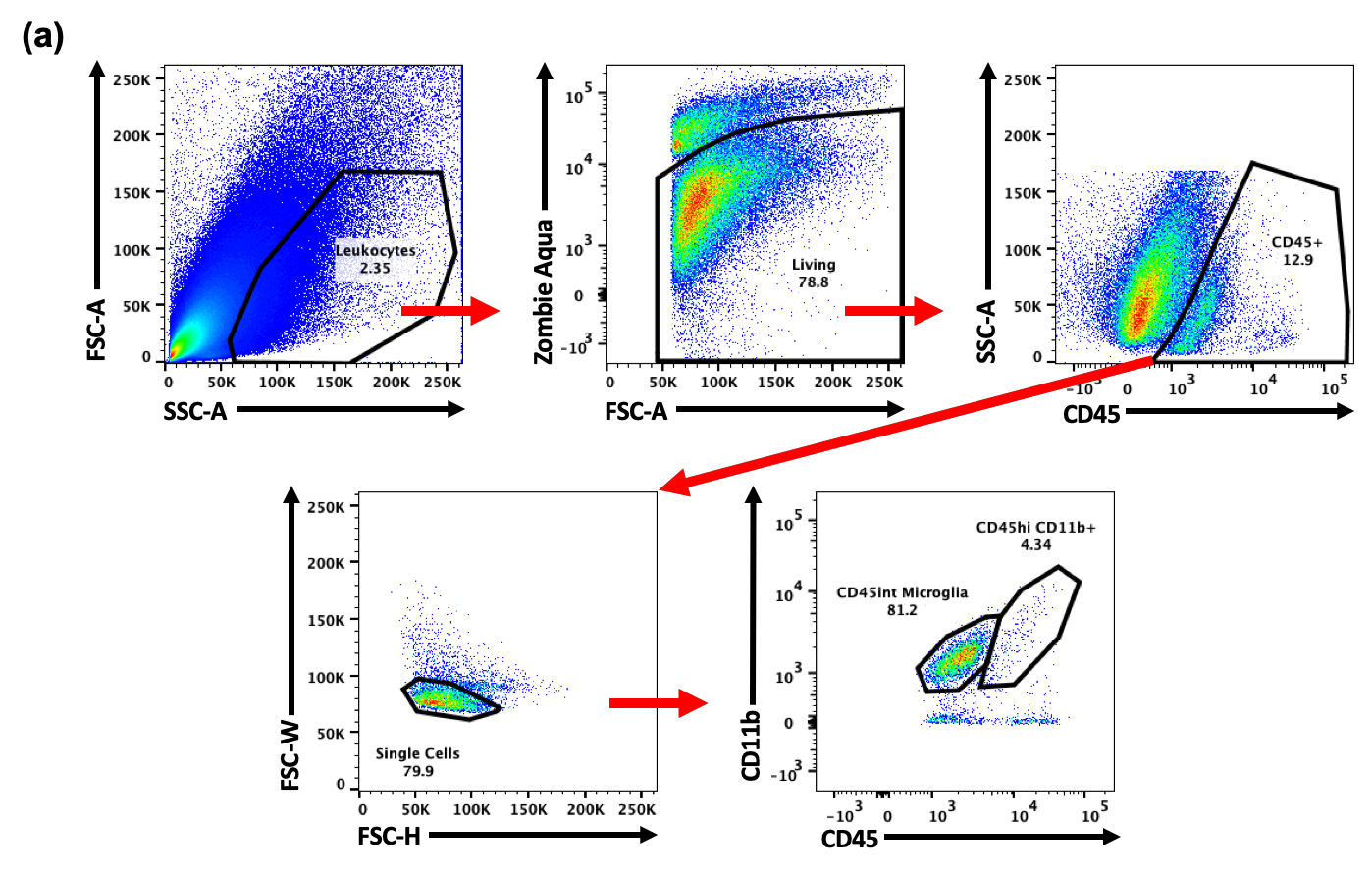
**

**SUPPORTING INFORMATION FIGURE S1. Flow cytometry gating strategy for brain leukocytes.** Brain cells were acquired on the cytometer and identified using the following gating strategy: putative leukocytes were gated using a splenocyte reference gate (FSC-A vs. SSC-A), living cells were gated using a viability dye (Zombie Aqua vs. FSC-A), white blood cells were identified using a pan-leukocyte marker (SSC-A vs. CD45), singlets were gated (FSC-W vs. FSC-H), and brain-resident microglia were identified as CD45^int^CD11b^+^ relative to the CD45^hi^CD11b^+^ myeloid cell population.

**
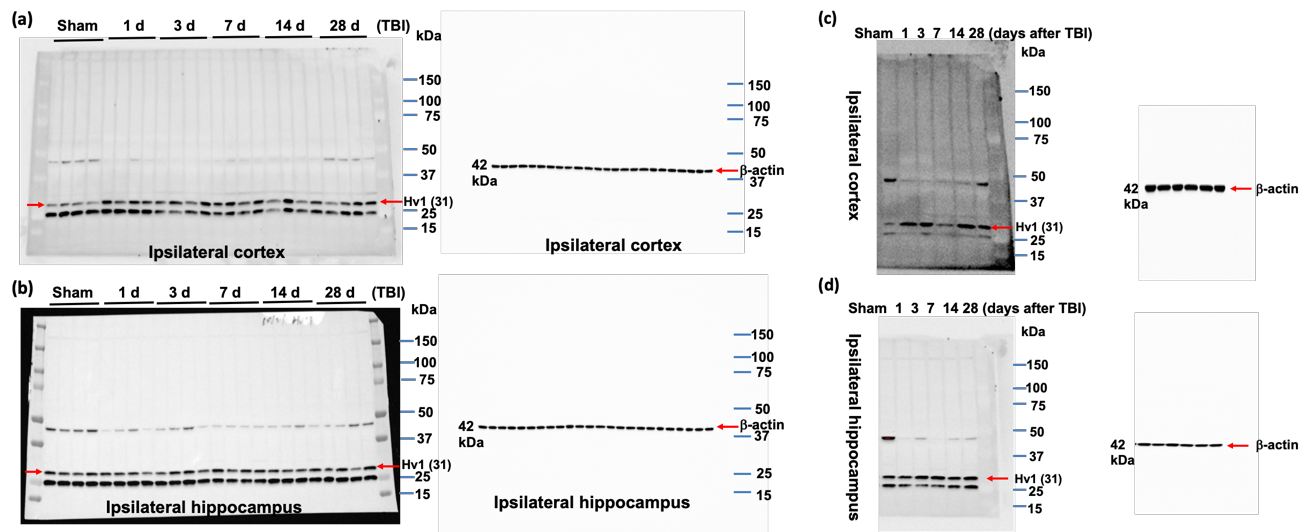
**

**SUPPORTING INFORMATION FIGURE S2. Western blot of Hv1 protein expression in the sham and TBI brain.** The full-length Western blot images for Figure 3h are shown. Hv1 protein expression (31 kDa band) in sham and day 1, 3, 7, 14, and 28 after TBI was determined based on normalization to the housekeeping protein β-actin (42 kDa band) in the ipsilateral (i.e., injury-side) (**a**) cortex and (**b**) hippocampus. The fifth sample in each group for Figure 3g is shown (**c**-**d**; n=5/grp). Abbreviations: KO knockout, WT wildtype.

**
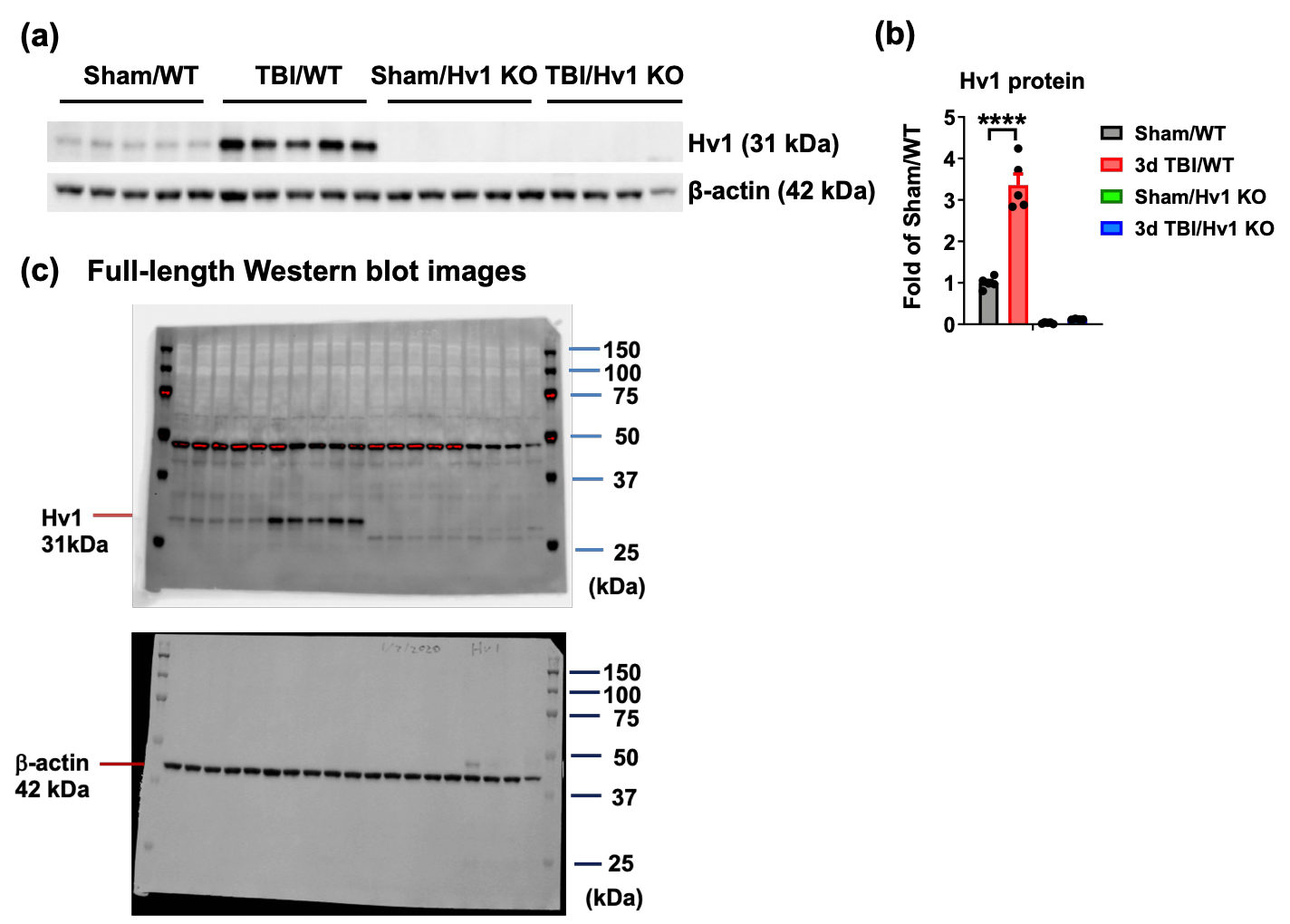
**

**SUPPORTING INFORMATION FIGURE S3. Western blot analysis of Hv1 protein expression in KO and WT mice.** Hv1 protein expression in WT and KO mice at 72 h after TBI was examined. (**a**) A representative Western blot image is shown. (**b**) Hv1 protein expression was normalized to β-actin and expressed as a fold-change to WT sham. The quantification shows the relative expression level of Hv1 increased significantly after TBI in WT mice but was absent in KO mice. (**c**) Full-length Western blot images for (**a**) are shown (n=5/group). Data in (**b**) were analyzed by two-way ANOVA using Tukey’s multiple comparison test to determine differences between sham and TBI (****p<0.0001). Data are mean ± SEM.


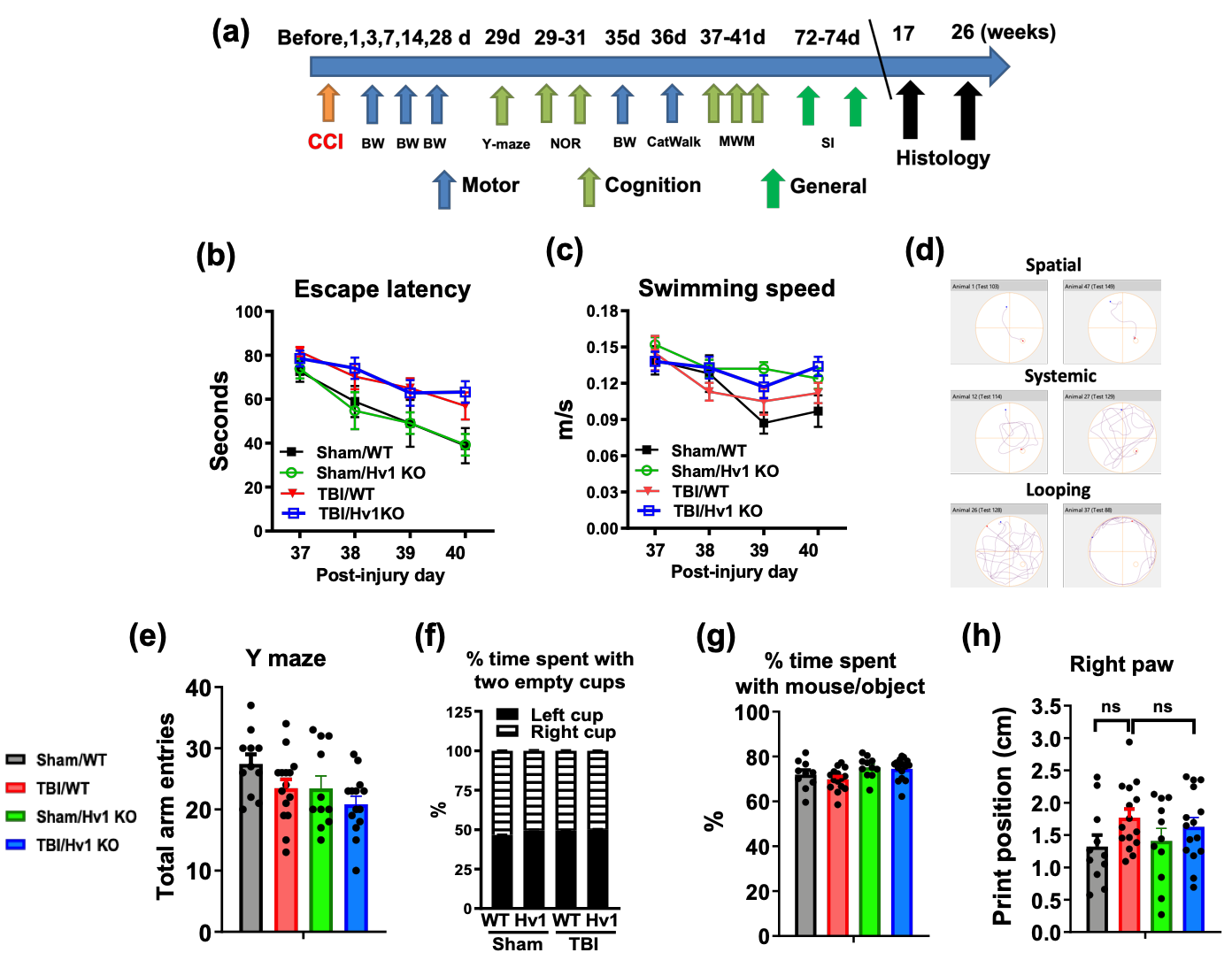


**SUPPORTING INFORMATION FIGURE S4. Diagram of behavioral assessment and phenotypic data.** (**a**) The behavioral test schedule is shown. Beam walk was examined before CCI and at 1, 3, 7, 14, 28, and 35 days; Y-maze at 29 days; novel object recognition at 29-31 days; the CatWalk at 36 days; Morris water maze at 37-41 days; and social interaction at 72-74 days post-injury. On weeks 17 and 26, all of the mice were euthanized and perfused for histology. No differences between genotype were seen in the (**b**) escape latency and (**c**) swimming speed in the Morris water maze task. (**d**) Representative images of search strategies used during the Morris water maze task are shown. (**e**) No difference in the number of arm entries was found between groups in the Y-maze. (**f**) The percentage of time spent with two empty cups during the first phase of the social interaction test shows no specific preference for the right or left chamber. (**g**) The percentage of time spent with a mouse versus an object in the second phase of the social interaction test was quantified. No differences were seen between groups. (**h**) Gait analysis was assessed using CatWalk. No statistical difference in the print position of the right paw was found in either genotype group after injury. For all behavioral experiments, n=11-15/group. Abbreviations: BW beam walk, CCI controlled cortical impact injury, KO knockout, NOR novel object recognition, ns not significant, m meters, MWM Morris water maze, s seconds, SI social interaction, WT wildtype. Data in (**b**-**c**) were analyzed by two-way ANOVA with repeated measures and Bonferroni’s test. Data in (**e**-**h**) were analyzed by two-way ANOVA using Tukey’s multiple comparison test to determine differences between sham and TBI and WT and KO TBI groups. Data are mean ± SEM.
